# Ultra-Sodiophilic Mixed Conductor Interphase Enabling Uniform Top Deposition for Quasi-Solid-State Sodium-Metal Batteries

**DOI:** 10.1007/s40820-026-02256-y

**Published:** 2026-06-29

**Authors:** Chunching Lu, Guangxiang Zhang, Yuxiang Niu, Yupeng Zhu, Siyuan Li, Hua Huo, Yulin Ma, Pengjian Zuo, Geping Yin, Yunzhi Gao, Liguang Wang, Chuankai Fu, Wei Chen

**Affiliations:** 1 State Key Laboratory of Space Power-Sources, School of Chemistry and Chemical Engineering, Harbin Institute of Technology, Harbin, 150001 People’s Republic of China; 2 Department of Chemistry, National University of Singapore, 3 Science Drive 3, Singapore, 117543 Singapore; 3 College of Chemical and Biological Engineering, Zhejiang University, Hangzhou, 310058 People’s Republic of China

**Keywords:** Quasi-solid-state, Sodium-metal batteries, Sodiophilic interphase, Ionic/electronic mixed conductor layer

## Abstract

Artificial solid electrolyte interphase offers a promising route to improve the lifespan and safety of quasi-solid-state sodium-metal batteries (QSMBs), yet its limited ion conductivity and the low liquidity of the polymer electrolyte often result in unstable Na plating/stripping kinetics and poor interfacial durability. Herein, we propose a surface-induced “top” Na deposition mechanism facilitated by an ultra-sodiophilic ionic/electronic mixed conductor interphase exhibiting strong Na^+^ adsorption affinity, which ensures fast and dendrite-free Na anode operation. The embedded Na_3_Sb alloy phase, featuring high electronic conductivity and strong Na^+^ adsorption energy, significantly accelerates interfacial ion diffusion and nucleation kinetics, forming a smooth and compact Na deposition layer that facilitates stable solid electrolyte interphase formation and preserves interfacial integrity. Consequently, Na||Na symmetric cells employing a 1, 3-dioxolane-based gel polymer electrolyte deliver an ultra-long-cycling lifespan of 1000 h at 0.5 mA cm^−2^ with a low overpotential of 40 mV. Moreover, QSMBs incorporating the modified Na anode and Na_3_V_2_(PO_4_)_3_ cathode demonstrate outstanding cycling stability (74.1% capacity retention after 9000 cycles at 2C) and superior rate capability (91.7 mAh g^−1^ at 5C). The work provides mechanistic insights and practical strategies for regulating Na deposition, paving the way toward high-performance QSMBs.

**Supplementary Information:**

The online version contains supplementary material available at 10.1007/s40820-026-02256-y.

## Highlights

- A surface-induced “top” Na deposition mechanism facilitated by an ultra-sodiophilic ionic/electronic mixed conductor interphase composed of NaF and Na_3_Sb is proposed.
- SFC-Na anode exhibits enhanced Na^+^ affinity force and accelerated ion transport kinetics, Na nucleation kinetics are greatly enhanced while dendrite growth is effectively suppressed.
- The quasi-solid-state sodium-metal batteries with Na_3_V_2_(PO_4_)_3_ cathode and SFC-Na anode exhibit significantly enhanced cycling stability and superior rate capability.

**Supplementary Information:**

The online version contains supplementary material available at 10.1007/s40820-026-02256-y.

## Graphical abstract

**Figure:**
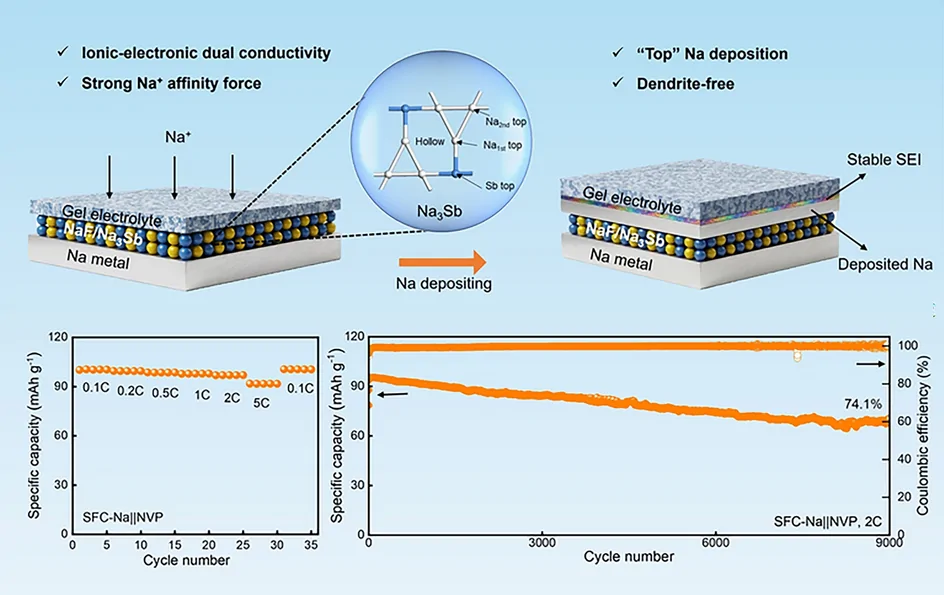

## Introduction

Sodium-metal batteries (SMBs) are promising candidates for next-generation energy storage, owing to the low cost, the abundance of sodium resources, and the competitive energy density [1–4]. However, repeated Na plating/stripping leads to uncontrolled dendritic growth, while the high flammability of conventional organic electrolytes poses serious safety risks [5, 6]. These issues collectively hinder the practical implementation of SMBs. Gel polymer electrolytes (GPEs), with enhanced thermal stability, high ionic conductivity, superior interface adhesion, and facile processability, offer a potential pathway to mitigate these hazards [5, 7, 8]. However, the local Na plating/stripping kinetics at the anode/electrolyte interface remains the decisive factor for achieving long-term and dendrite-free operation. The surface physicochemical properties of Na anode play a critical role in determining the interfacial distribution of charge and Na^+^. However, the naturally formed solid electrolyte interphase (SEI) on the Na anode generally suffers from poor mechanical integrity, compositional heterogeneity, and low ionic conductivity [9, 10]. These limitations render the spontaneous SEI inadequate for long-term stable SMBs. Therefore, constructing an ideal artificial solid electrolyte layer (ASEI) has emerged as a crucial strategy to regulate Na deposition and suppress dendrite growth at the interface.

Common approaches for constructing an ASEI encompass electrolyte composition optimization, physical coatings, magnetron sputtering, and atomic layer deposition [11, 12]. The core objective of these methods is to create an ionic-conducting yet electronically insulating interlayer, which effectively blocks undesired electron transfer across the interface. For instance, Chen et al. [13] enhanced the long-term stability of the anode/electrolyte interface by forming an anion-derived inorganic-rich SEI layer. However, while an ionic-conductive interphase (ICI) can effectively inhibit electron transfer, its inherent rigidity often leads to poor contact with non-flowing GPEs, resulting in non-dense Na deposition. During cycling, the repeated Na^+^ flux through the ICI for electrochemical deposition and dissolution can induce mechanical fracture and physical degradation of the layer [14]. Such failures subsequently disrupt ion transport, accelerate dendrite penetration, and perpetuate interfacial side reactions. Moreover, excessive accumulation of inorganic substances can lead to an increase in the Na^+^ diffusion energy barrier, adversely affecting the rate capability of SMBs [15].

Herein, we propose a surface-induced “top” Na deposition paradigm based on a multifunctional ionic/electronic mixed conductor interphase (MCI) formed in situ on the Na anode by the controlled reaction of SbF_3_ with Na metal, yielding a composite primarily composed of NaF and Na_3_Sb alloy. Owing to the high intrinsic chemical stability, sodiophilicity, electronic conductivity, and exceptional Na^+^ conductivity of MCI, the MCI surface serves as the preferred site for Na^+^ reduction. This unique combination of properties drives a dense, conformal Na deposition and promotes the formation of a compact and uniform SEI. Multidimensional physical characterization, theoretical calculations, and electrochemical testing confirm this unique surface-induced “top” Na deposition mechanism of the MCI. Consequently, optimized Na||Na symmetric cells with 1,3-dioxolane-based gel polymer electrolyte (DGPE) demonstrate significantly enhanced cycling stability, while the quasi-solid-state SMBs (QSMBs) employing a Na_3_V_2_(PO_4_)_3_ cathode exhibit superior rate capability, prolonged cycle life, and stable Coulombic efficiency (CE).

## Experimental Section

### Materials

Na_3_V_2_(PO_4_)_3_ (NVP) cathode, Na foils (thickness of 400 µm), conductive carbon, and glass-fiber (GF/D, Whatman) separators are commercially available. Battery-grade sodium hexafluorophosphate (NaPF_6_, 99%), 1,3-dioxolane (DOL), methoxymethane (DME), fluoroethylene carbonate (FEC), carboxymethyl cellulose (CMC), polyvinylidene fluoride (PVDF), and antimony fluoride (SbF_3_) were purchased from Aladdin.

### Preparation of Electrodes and Polymer Electrolyte

#### Preparation of Polymer Electrolyte

The liquid electrolyte was prepared by dissolving a certain amount of NaPF_6_ salts into a mixed solvent (DOL: DME: FEC = 1:0.8:0.2 by vol) with a fixed molar concentration of 1 mol L^−1^. The liquid electrolyte is injected into the glass-fiber separator, which can be converted into the quasi-solid-state electrolyte in situ at room temperature.

#### Preparation of F–Na and SFC-Na Anodes

SbF_3_ was dissolved in DME with a certain concentration and vigorously stirred for several hours until fully dissolved. Subsequently, the Na foils were immersed for 180 s to complete the reaction. The treated Na foils were washed with DME and dried overnight in a glove box designated as SF-Na. In addition, a small amount CMC binder was added to the SbF_3_ precursor solution before immersing Na foils to boost the mechanical strength of the resulting protective layer named SFC-Na. The F-Na anode was prepared by immersing the Na anode in pure fluorinated ethylene carbonate (FEC) solvent for 5 h.

#### Preparation of NVP Cathode

The NVP active materials, PVDF binder, and conductive carbon were mixed into a homogeneous slurry with a weight ratio of 7: 2: 1 and then cast onto an Al foil current collector and dried in a vacuum oven at 120 °C for 12 h. The coated electrodes were cut into 14-mm-diameter disks with a mass loading of about 2 and 5 mg cm^–2^.

#### Fabrication of Na||Na Symmetric Cells and Na||NVP Cells

The Na||Na symmetric cells were assembled in the standard CR2025 coin-cell with pristine Na, F–Na, SFC-Na, GF/D separators, and a certain amount of electrolytes (about 120 μL). The Na||NVP cells were assembled in the standard CR2025 coin-cell with the as-prepared anodes (pristine Na, F-Na, and SFC-Na), NVP cathode, GF separators, and a certain amount of the as-prepared electrolytes (about 120 μL). The commercial NVP cathode with a high mass loading of 20 mg cm^−2^, provided by Harbin Coslight Power Co., Ltd., was used to assemble 200 mAh-class pouch cells. The electrolyte usage was 2.5 g Ah^−1^. All cells were assembled in a glove box with H_2_O and O_2_ below 0.01 ppm. The electrolyte in the assembled cells can change from liquid to solid at room temperature after 2 h.

### Electrochemical Testing and Physical Characterization

#### Electrochemical Testing

The Na||Na symmetric cells were subjected to cyclic tests of charging for one hour and discharging for one hour at different current densities. The charging/discharging tests of Na||NVP cells were assembled and performed in the potential range of 2.5–3.6 V at different current densities. The electrochemical impedance spectra (EIS) of Na||Na symmetric cells and Na||NVP cells before and after cycling were studied on a CHI 660E electrochemical workstation in the frequency range of 10^5^ ~ 0.1 Hz with an amplitude of ± 10 mV. The Tafel curves were obtained by measuring the Na||Na symmetric cell by the LSV method with 0.5 mV s^−1^. According to the Tafel Eq. (1):

1 $$\eta = a + b \times \log \left( I \right)$$

The plots of log(*I*) vs. *η* can be obtained. Then, the exchange current density (log(*i*_0_)) can be calculated by extrapolating the linear part to *η* = 0 [16].

#### Physical Characterization

The samples’ morphology and elemental distribution were characterized by high-resolution field emission scanning electron microscopy (SEM, Helios Nanolab 600i) equipped with an EDS analyzer with an acceleration voltage of 20 kV. The X-ray diffraction (XRD) of SFC-Na was performed using Cu-Kα radiation, with samples sealed using a Kapton-type seal to mitigate air interference during testing. X-ray photoelectron spectroscopy (XPS, ESCALAB 250Xi) was conducted on a PHI model 5700 instrument with the Al-Kα source to analyze the chemical composition of different Na anode surfaces. The electronic conductivity of Na_3_Sb and NaF/Na_3_Sb is obtained through a resistivity tester. The Raman spectra were obtained by LabRAM HR Evolution (785 nm laser). Solid-state nuclear magnetic resonance (NMR) spectroscopy (Bruker 400 MHz AVANCE III spectrometer) and gel permeation chromatography (GPC) were used to analyze the polymerization behavior of electrolytes. The X-ray imaging experiments were carried out at the Shanghai Synchrotron Radiation Facility of BL13HB.

### Theoretical Calculation and Phase-Field Simulation

#### Theoretical Calculation

Surface and adsorption energy were calculated using density functional theory (DFT) by Materials Studio. The Perdew–Burke–Ernzerhof (PBE) generalized gradient approximation (GGA) served as the exchange–correlation generalized function. The structures employed were structurally optimized and assessed for truncation energy convergence [17].

For surface energy, in examining the adsorption of Na^+^ on the surfaces of both NaF and Na_3_Sb, it is essential to identify a stable surface (characterized by low Miller indices) to serve as the adsorption interface. The surface energy can be determined using the following formula Eq. (2):

2 $$\gamma = \left( {E_{{{\mathrm{surf}}}} - nE_{{{\mathrm{bulk}}}} } \right)/2S$$

Of which, $E_{{{\mathrm{surf}}}}$ is the energy of the plane, $E_{{{\mathrm{bulk}}}}$ is the energy of a single atom or the smallest unit in the block structure, n is the number of atoms or smallest units in the plane, and *S* is the surface area of the plane. The results of the calculations are shown in Figs. S6, S7, and Table S1, and the crystal planes with the lowest NaF surface energies are (001) planes (0.16 eV Å^−2^) and (110) planes (0.21 eV Å^−2^) for Na_3_Sb [18]. Therefore, NaF (001) and Na_3_Sb (110) are theoretically employed as the respective adsorption surfaces. The adsorption energy of Na^+^ on NaF and Na_3_Sb can be calculated as follows:

3 $$E_{{{\mathrm{ads}}}} = \frac{{E_{{{\mathrm{gra}}}} + nE_{i} - E_{{{\mathrm{tot}}}} }}{n}$$

where $E_{{{\mathrm{ads}}}}$ is the adsorption energy, $E_{{{\mathrm{gra}}}}$ is the total energy of the system without adsorbed atoms, $E_{{{\mathrm{tot}}}}$ is the total energy of the system with adsorbed atoms, $E_{i}$ is the energy of a single adsorbed atom, and n is the number of adsorbed atoms.

#### Phase-Field Simulation

The phase-field simulation was conducted using COMSOL software. The multi-physical field simulation software employs a general PDE, configuring the solver for time-dependent analysis. Dirichlet boundary conditions were applied for the Nernst–Planck equation and the current continuity equation; for the phase-field variable $\left( \xi \right)$, zero flux boundary conditions were established. The concentration of C_Na+_ in the electrolyte was fixed at 1.0 mol L^–1^, while in the electrode it was set to 0.0 mol L^–1^. The boundary conditions were defined with the electrode potential (*φ*) at 0 V and the electrolyte potential (*φ*) at 0.5 V to investigate the impact of an artificial protective layer on electrode performance, particularly in the morphology of sodium deposition. A highly diffusive solid electrolyte interphase (SEI) layer was introduced on the surface of the Na anode to simulate the processed sodium [19]. The corresponding computational models and detailed calculations are provided below.

The growth of Na dendrites can be characterized by the temporal progression of the phase-field variable *ξ*, achieved by resolving the subsequent governing Eq. (4):

4 $$\frac{\partial \xi }{{\partial t}} = - L_{\sigma } \left( {g^{\prime}\left( \xi \right) - k\nabla^{2} \xi } \right) - L_{\eta } h^{\prime}\left( \xi \right)\left\{ {\exp \left[ {\frac{\alpha zF\eta }{{RT}}} \right] - C\exp \left[ { - \frac{\beta zF\eta }{{RT}}} \right]} \right\}$$

In this context, *t* represents time, *L*_*σ*_ denotes interfacial mobility, and *L*_*η*_ signifies the electrodeposition reaction constant. The term $g^{\prime}(\xi )$ refers to the first derivative of the double well function defined as $g(\xi ) \, = W\xi^{2} (1 \, - \xi )^{2}$, where *W* indicates the barrier height. The parameter κ is the gradient energy coefficient, while $h^{\prime}(\xi )$ represents the first derivative of the interpolating function $h(\xi ) \, = \xi^{3} (6\xi^{2} - \, 15\xi + \, 10)$. The symmetric factors α and β satisfy the condition *α* + *β* = 1. Additionally, *z* denotes the charge number of the sodium ion, *F* is the Faraday constant, *η* represents the overpotential, *R* is the universal gas constant, and *T* is the absolute temperature. The temporal dynamics of the sodium ion concentration (C_Na⁺_) are derived by solving the Nernst–Planck equation:

5 $$\frac{\partial \xi }{{\partial t}} = \nabla \left[ {D^{{{\mathrm{eff}}}} \nabla c_{{{\mathrm{Li}}}} + \mu_{{{\mathrm{Li}}}} c_{{{\mathrm{Li}}}} zF\nabla \psi } \right] - K\frac{\partial \xi }{{\partial t}}$$

where *D*^*eff*^ is the effective Li ion diffusivity. The constant *K* signifies the accumulation factor. Under the assumption of charge neutrality within the system, the conservation of current density is articulated through the current continuity equation, $\nabla \left( {\sigma^{eff} \nabla \psi } \right) = R\frac{{\partial {\upxi }}}{\partial t}$, where σ^*eff*^ is the effective electrical conductivity of the system. *R* = *csz*η*F* represents the current constant for the source term, which quantifies the charge influx or efflux within the system as a result of chemical reactions, where *cs* denotes the site density of Na metal [20].

## Results and Discussion

### Design and Preparation of ICI and MCI

**Fig. 1:**
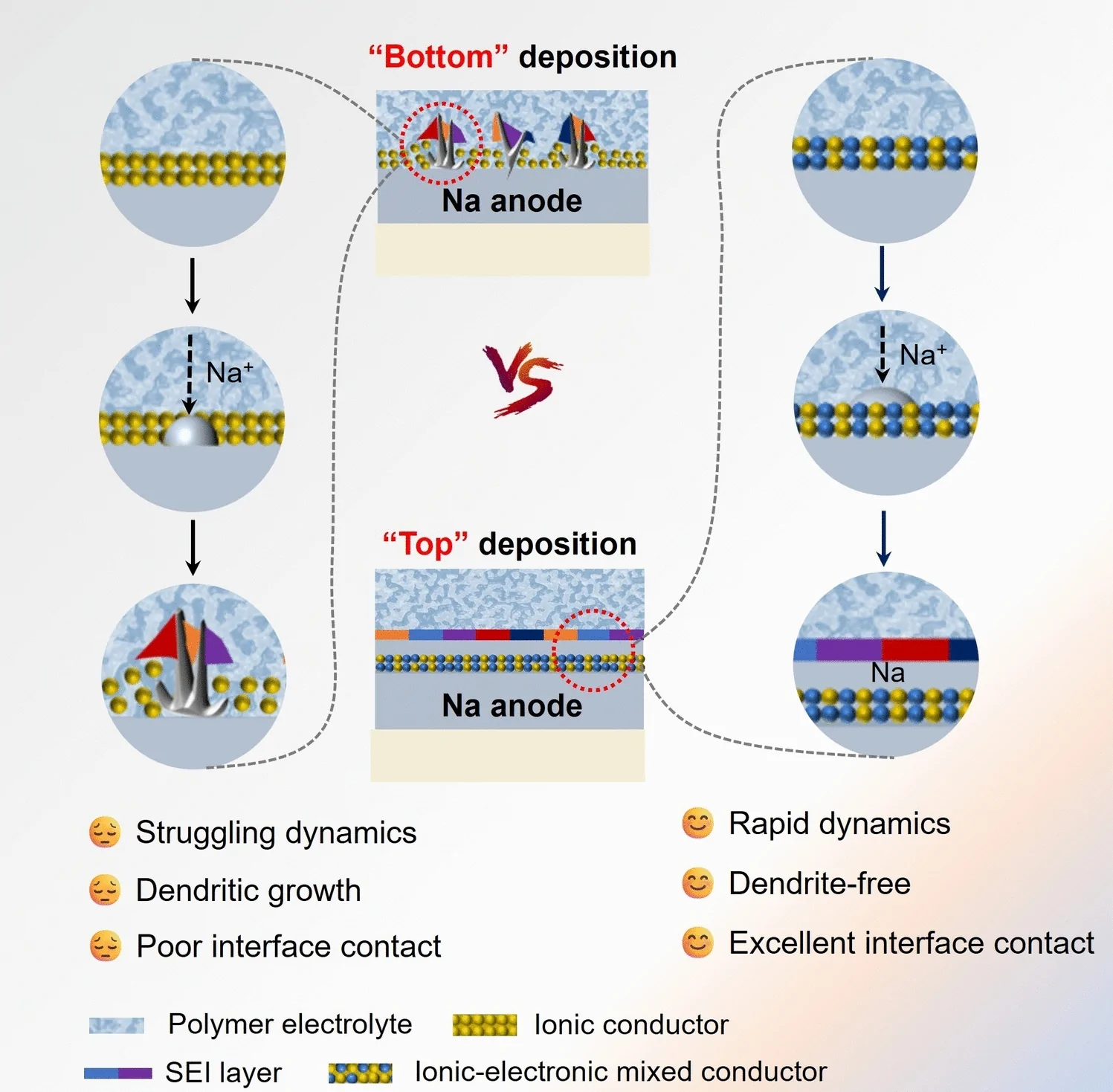
Schematic illustration of Na plating/stripping models

As shown in Fig. 1, the conventional ICI exhibits solely Na^+^ conductivity, which helps suppress undesirable interfacial charge and energy transfer, consistent with the “bottom” deposition model. However, the intrinsic rigidity of the ICI, combined with the poor fluidity of the GPEs, leads to non-uniform Na plating/stripping during cycling. This readily causes mechanical fracture and physical damage to the ICI, consequently inducing dendrite growth and penetration, as well as poor interface contact. In contrast, the MCI with electronic conductivity enables the reduction of Na^+^ to metallic Na on its surface, effectively preventing structure failure of MCI during repeated Na plating/stripping processes, in accordance with the surface-induced “top” deposition model. Furthermore, the dense Na deposition layer on the top of MCI contributes to the formation of a complete and uniform SEI layer, thereby enhancing long-term interfacial stability.

**Fig. 2:**
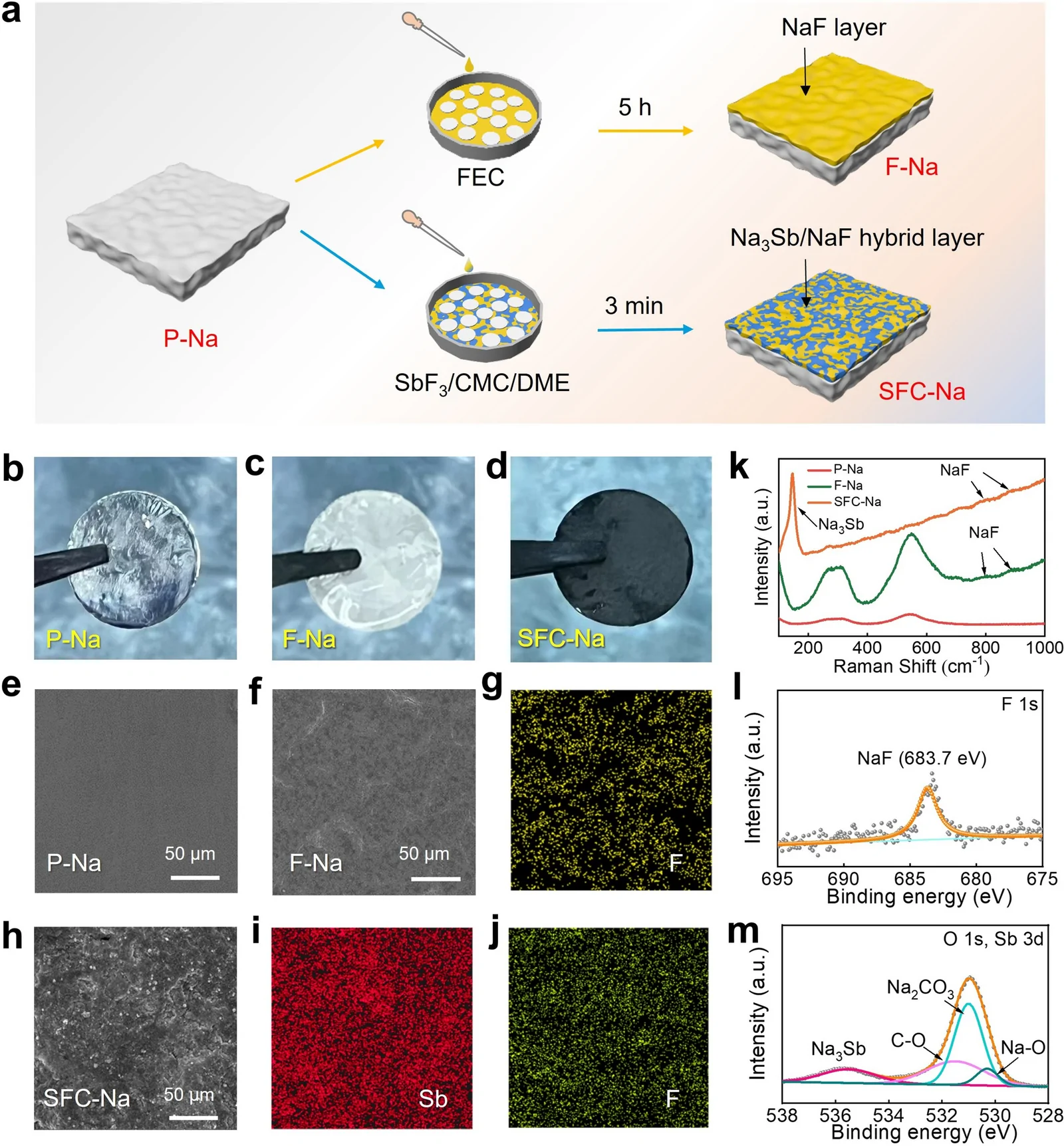
Preparation and characterization of the F-Na and SFC-Na. **a** The synthesis diagram of the F-Na and the SFC-Na. **b**–**d** Optical photographs of P-Na, F-Na, and SFC-Na. SEM images of P-Na **e**, F-Na **f**, and SFC-Na **h**, corresponding element mapping of F-Na **g** and SFC-Na **i, j**. **k** Raman spectra of the F–Na and SFC-Na. XPS spectra of **l** F 1*s* and **m** O 1*s* of the SFC-Na

The preparation processes of the ICI and MCI are illustrated in Fig. 2a. A typical ICI layer composed solely of NaF was constructed on the Na surface by immersing the Na anode in pure fluorinated ethylene carbonate (FEC) solvent for 5 h. In contrast, the MCI was fabricated by immersing the Na anode in a chemical treatment solution containing SbF_3_ in 1,2-dimethoxyethane (DME) for 3 min. Driven by the chemical potential between SbF_3_ and Na metal, a heterogeneous MCI rich in NaF and Na_*x*_Sb alloy spontaneously forms on the surface of the Na anode, described by the following chemical reaction Eqs. (1) and (2) [21]. Based on the surface reaction uniformity (Fig. S1), stability of Na plating/stripping (Fig. S2), and solubility of SbF_3_ in DME solvent (Fig. S3), the optimal concentration of SbF_3_ can be determined to be 0.1 mol L^–1^. To enhance the mechanical stability of the MCI, a small amount CMC binder is incorporated into the chemical treatment solution to construct a cross-linked network. Considering the long-term stability of the artificial interphase layer, the dosage of CMC binder was fixed at 1 wt% based on a single factor experiment (Fig. S4). In addition, adhesion tests show that the addition of 1 wt% CMC effectively promotes the adhesion between the MCI layer and the Na metal substrate (Fig. S5). The anodes treated by FEC and SbF_3_ are marked as F-Na and SFC-Na, respectively, while the pristine Na anode is marked as P-Na. As shown in Fig. 2b–d, distinct color changes can be observed on the surface of the Na anodes after the chemical treatment: silver for P-Na, milky white for F-Na, and black for SFC-Na.

6 $$3{\mathrm{Na}} + {\mathrm{SbF}}_{3} \to {\mathrm{Sb}} + 3{\mathrm{NaF}}$$

7 $$x{\mathrm{Na}} + {\mathrm{Sb}}\user2{ } \to {\text{ Na}}_{x} {\mathrm{Sb}}$$

The micromorphology and chemical components of F-Na and SFC-Na anodes were characterized by scanning electron microscopy (SEM), Raman spectra, X-ray diffraction (XRD), and X-ray photoelectron spectroscopy (XPS). As shown in Fig. 2e, f, and h, the F–Na surface is covered with a dense and smooth protective layer, whereas the SFC-Na anode exhibits a relatively rough surface comprised of solid particles. As shown in Fig. 2g, i, and j, both the F element on F-Na and Sb and F elements on SFC-Na are uniformly distributed. The Raman spectrum of F-Na shows a distinct characteristic peak of NaF, while NaF and Sb_3_Na alloy can also be observed in the Raman spectrum of SFC-Na (Fig. 2k). Meanwhile, as shown in F 1*s* and Sb 3*d* XPS spectra (Fig. 2l, m), NaF (683.7 eV) and Na_3_Sb (535.5 eV) alloy signals can be observed on the SFC-Na anode [2]. In addition, the C=O–OR, C–O, and other organic components generated by DME solvent can improve the flexibility of the MCI (Figs. 2m and S6) [22]. The presence of organic components and NaF is also confirmed on F-Na (Fig. S7). XRD analysis further verifies the phase composition, with distinct diffraction peaks corresponding to NaF (PDF#70–2508) and Na₃Sb alloy (PDF#74–1162) in the surfaces of F-Na and SFC-Na anodes (Fig. S8) [23–25]. Surprisingly, owing to the high compactness and excellent chemical stability of MCI, the SFC-Na anode can remain stable for 30 min in the air, which is superior to that of F-Na (3 min) and P-Na (5 min) (Fig. S9). Excellent air stability is favorable for the practical application of the metal Na anode.

### Interfacial Na^+^ Dynamics and Thermodynamic Behaviors

**Fig. 3:**
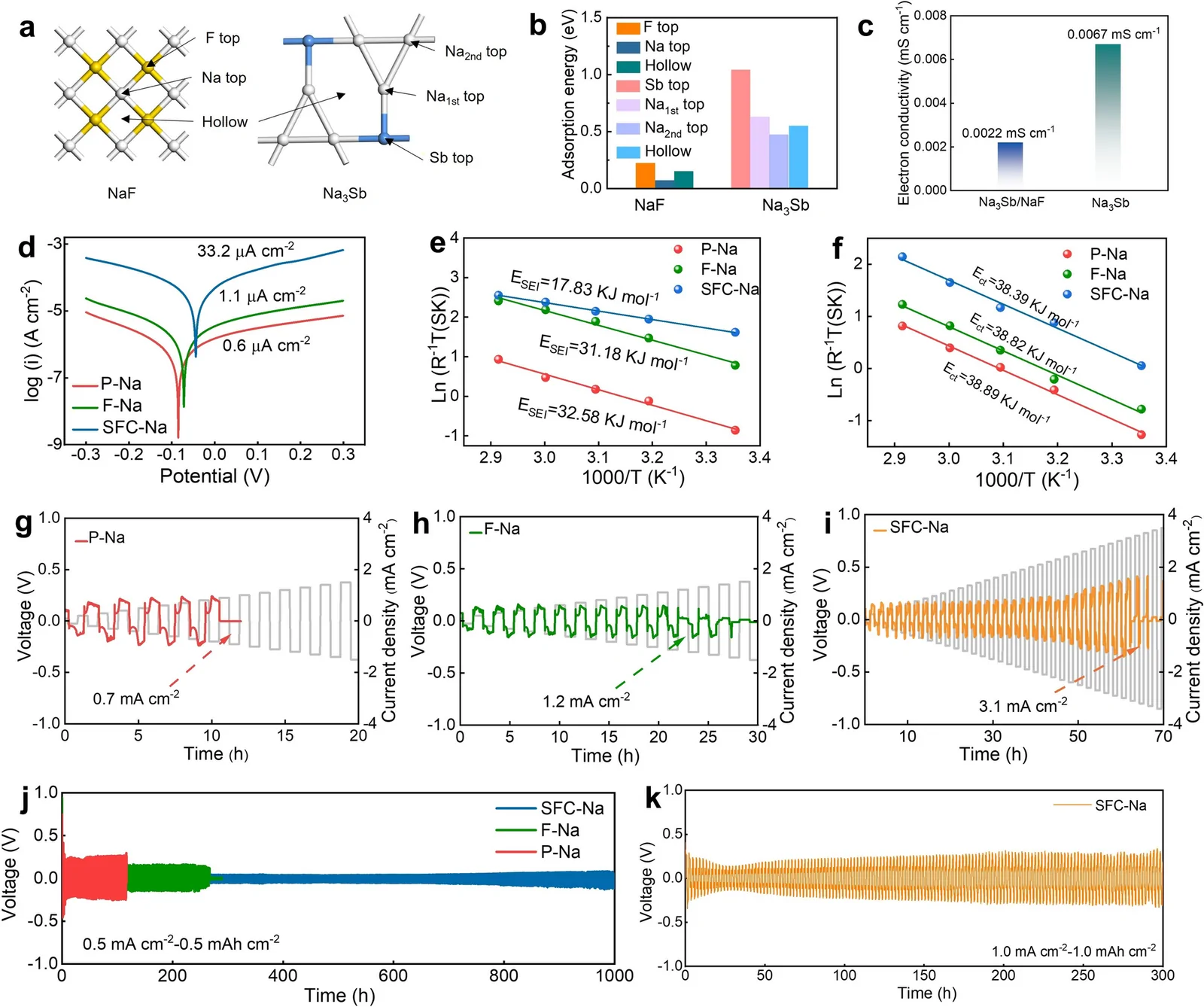
Interfacial Na^+^ transport and electrochemical performance of Na||Na symmetric cells. **a** Adsorption sites and **b** corresponding adsorption energies of NaF and Na_3_Sb with Na^+^. **c** Electron conductivity of Na_3_Sb and Na_3_Sb/NaF. **d** Tafel plot of symmetrical cells with P-Na, F-Na, and SFC-Na anodes. **e, f** Activation energies of Na^+^ transport through the SEI layer and charge-transfer process. The CCD tests of P-Na **g**, F-Na **h**, and SFC-Na **i** anodes. **j** Long-cycling performance of Na||Na symmetric cells with P-Na, F-Na, and SFC-Na anodes at 0.5 mA cm^−2^. **k** Long-cycling performance of Na||Na symmetric cells with P-Na, F-Na, and SFC-Na anodes at 1.0 mA cm^−2^

The kinetic properties of interfacial Na⁺ transport and charge transfer are systematically investigated via density functional theory (DFT), Tafel plots, and electrochemical impedance spectroscopy (EIS). The evolution mechanism of Na deposition on the MCI is elucidated by DFT, using modeled Na₃Sb (110) and NaF (001) surfaces with the lowest surface energies (Figs. S10, S11, and Table S1) [18]. Calculation of Na⁺ adsorption energies across various sites on these surfaces (Fig. 3a) identifies the Sb-top of Na₃Sb as the most stable, with an energy of 1.07 eV, and the F-top of NaF (001) as the most favorable, at 0.23 eV. Obviously, the adsorption energies of Na^+^ on Na_3_Sb are all greater than that on NaF (Fig. 3b), indicating that Na_3_Sb has a higher affinity for Na^+^. The diffusion process of Na^+^ in NaF and Na atom in Na_3_Sb is simulated. As shown in Fig. S12, the diffusion energy barrier of Na^+^ in NaF is 0.704 eV, which is much higher than Na atom in Na_3_Sb (0.0609 eV), demonstrating the significant role of Na_3_Sb in promoting electrochemical kinetics. Furthermore, the band gap calculation results (Fig. S13) show that the band gap of NaF is as high as 6.06 eV, while Na_3_Sb is a semi metallic phase with a band gap of only 0.339 eV. Therefore, NaF in MCI exhibits high electron blocking ability, which can effectively prevent electron tunneling, while Na_3_Sb phase can improve electron conductivity, achieve rapid electron transfer, and avoid charge accumulation. Notably, the MCI demonstrates considerable electronic conductivity (0.0022 mS cm^−2^), facilitating efficient Na plating/stripping (Fig. 3c).

Symmetrical cells employing DOL-based gel polymer electrolyte (DGPE), synthesized via in situ polymerization initiated by NaPF₆ at room temperature, are assembled for EIS testing. As shown in Fig. S14, the polymerization mechanism involves PF_5_-a Lewis acid derived from NaPF_6_-reacting with trace water to generate protons that initiate the ring-opening polymerization of DOL [26]. The ^13^C-NMR spectrum further confirmed the polymerization process, where the chemical shift of carbon atoms in DOL shifted from the original 94.5 and 64.2 ppm to 95.2 and 66.8 ppm (Fig. S15a), indicating successful polymerization of DOL [27, 28]. Furthermore, the polymerization degree of DGPE can be calculated based on ^1^H-NMR results, as follows:

8 $$n = 100\% \times {\mathrm{Hp}}/\left( {{\mathrm{Hp}} + {\mathrm{Hm}}} \right)$$

where n is the polymerization degree, H_p_ is the integrated area of the H atoms peaks of poly-ether chain in 1H-NMR spectrum (Fig. S15b), and H_m_ is the integrated area of the H atoms peaks of DOL monomer. Then, the polymerization degree of DGPE is calculated to be 89.7%. In addition, the number-average molecular weight and weight-average molecular weight of DGPE obtained by gel permeation chromatography (GPC) are 5149 and 27,616 g mol^–1^, respectively (Fig. S15c). In addition, the impedance of the Na||Na symmetric cells before cycling remain high stability after long-term storage (Fig. S16), revealing the high chemical compatibility between DGPE electrolyte and SFC-Na anode.

The Tafel plot is conducted to obtain the exchange current density (*i*_0_) of the Na||Na symmetric cells to reflect the transfer kinetics of interfacial Na^+^ (Fig. 3d) [16]. Compared to the P-Na (0.6 μA cm^−2^) and F-Na anodes (1.1 μA cm^−2^), the SFC-Na anode possesses a higher *i*_0_ (33.2 μA cm^−2^), indicating that the MCI can facilitate the Na plating/stripping and charge transfer. Furthermore, the impedances of Na^+^ transport across the SEI (*R*_SEI_) layer and charge transfer (*R*_ct_) at different temperatures are further measured by EIS to reflect the superiority of the MCI in inducing the construction of the stable and robust SEI layer and interfacial Na^+^ migration (Fig. S17). The EIS values are fitted by the corresponding equivalent circuit (Fig. S18). Figure S19 shows that the *R*_SEI_ and *R*_ct_ of quasi-solid-state symmetrical cells with F-Na and SFC-Na anodes are all far lower than those with P-Na at various temperatures, indicating the superiority of interface protection strategy. In addition, the quasi-solid-state symmetrical cells with SFC-Na anode possess the lowest *R*_SEI_ and *R*_ct_, demonstrating the improved interfacial stability between SFC-Na anode and DGPE gel electrolyte. Arrhenius Eq. (9) can quantify the activation energy of Na^+^ transport through the anode/electrolytes interface:

9 $$\ln \left( {\frac{T}{{R_{{{\mathrm{EIS}}}} }}} \right) = - \frac{{E_{a} }}{R*T} + \ln A$$

where *T*, *R*_EIS_, *R*, and *A* represent absolute temperature, impedance, standard gas constant, and preexponential constant, respectively [29, 30].

Based on EIS data at different temperatures, the activation energy of different anodes is calculated and shown in Fig. 3e, f. Benefitting from the rapid interfacial ion–electron transfer, the activation energy of Na^+^ transport through the SEI (*E*_SEI_) of the SFC-Na anode is 17.83 kJ mol^−1^, which is much smaller than that of the P-Na anode (32.58 kJ mol^−1^) and the F-Na anode (31.18 kJ mol^−1^), demonstrating the superiority of MCI in constructing stable SEI layer. Moreover, the activation energy of charge-transport (*E*_ct_) of the SFC-Na anode (38.39 kJ mol^−1^) is relatively smaller than that of the P-Na anode (38.89 kJ mol^−1^) and the F-Na anode (38.82 kJ mol^−1^). These reduced energy barriers confirm the excellent synergy effect of NaF and Na_3_Sb in regulating Na⁺ diffusion and interfacial charge transfer. Together, these results demonstrate that the MCI enhances Na plating/stripping kinetics and promotes the long-term interfacial stability.

To verify the superiority of the SFC-Na anode, the galvanostatic long-term discharging performance of Na||Na symmetric cells fabricated by the pristine anode (P-Na) and the modified anodes (F-Na and SFC-Na anodes) is measured at 0.5 mA cm^–2^ [31]. As shown in Fig. S20, the cells with P-Na and F-Na stabilized for only 2 and 8 h, respectively, before short circuit, whereas the SFC-Na cell exhibits a stable voltage plateau at 80 mV for 22 h and minimum polarization voltage, demonstrating effective suppression of Na dendrite growth and rapid Na nucleation dynamics, which is consistent with the calculation results of the diffusion energy barrier. Furthermore, the current density of the Na dendrite penetrating the polymer electrolyte is referred to as the critical current density (CCD), which is a crucial parameter for evaluating anode resistance to dendrite growth [32]. Figure 3g–i evinces the CCD of the Na||Na symmetric cells with various anodes, where the cells undergo an initial cycling process of 50 cycles at a current density of 0.1 mA cm^−2^, followed by a gradual escalation of the current density. The results indicate that the CCD of the P-Na symmetric cell is merely 0.6 mA cm^−2^, whereas the SFC-Na symmetric cell achieves a CCD of 3.1 mA cm^−2^, demonstrating that the artificial MCI effectively broadens the operational current density. The cycling stability of the symmetric cells with various anodes is further evaluated at 0.5 mAh cm^−2^. The SFC-Na anode with 1wt% CMC binder achieves stable cycling for 1000 h (Fig. 3j), substantially outperforming P-Na (100 h), F-Na (200 h), and other SFC-Na anodes with different CMC contents (Fig. S21), indicating that the cross-linked network formed by CMC binder promotes more stable Na plating/stripping behavior. Even at higher current densities of 1 and 2 mA cm^−2^, the SFC-Na cells cycled stably for 300 and 180 h, respectively, without short circuit (Figs. 3k and S22). The superior stability and interfacial compatibility of the SFC-Na anode stand out among the reported findings concerning artificial protective layers (Table S2) [9, 33–40]. These findings demonstrate that the MCI composed of NaF and Na_3_Sb can facilitate Na^+^ diffusion and contribute to uniform, flat Na deposition, enabling a prolonged lifespan.

### Unique Na Plating/Stripping Behavior on the MCI Surface

**Fig. 4:**
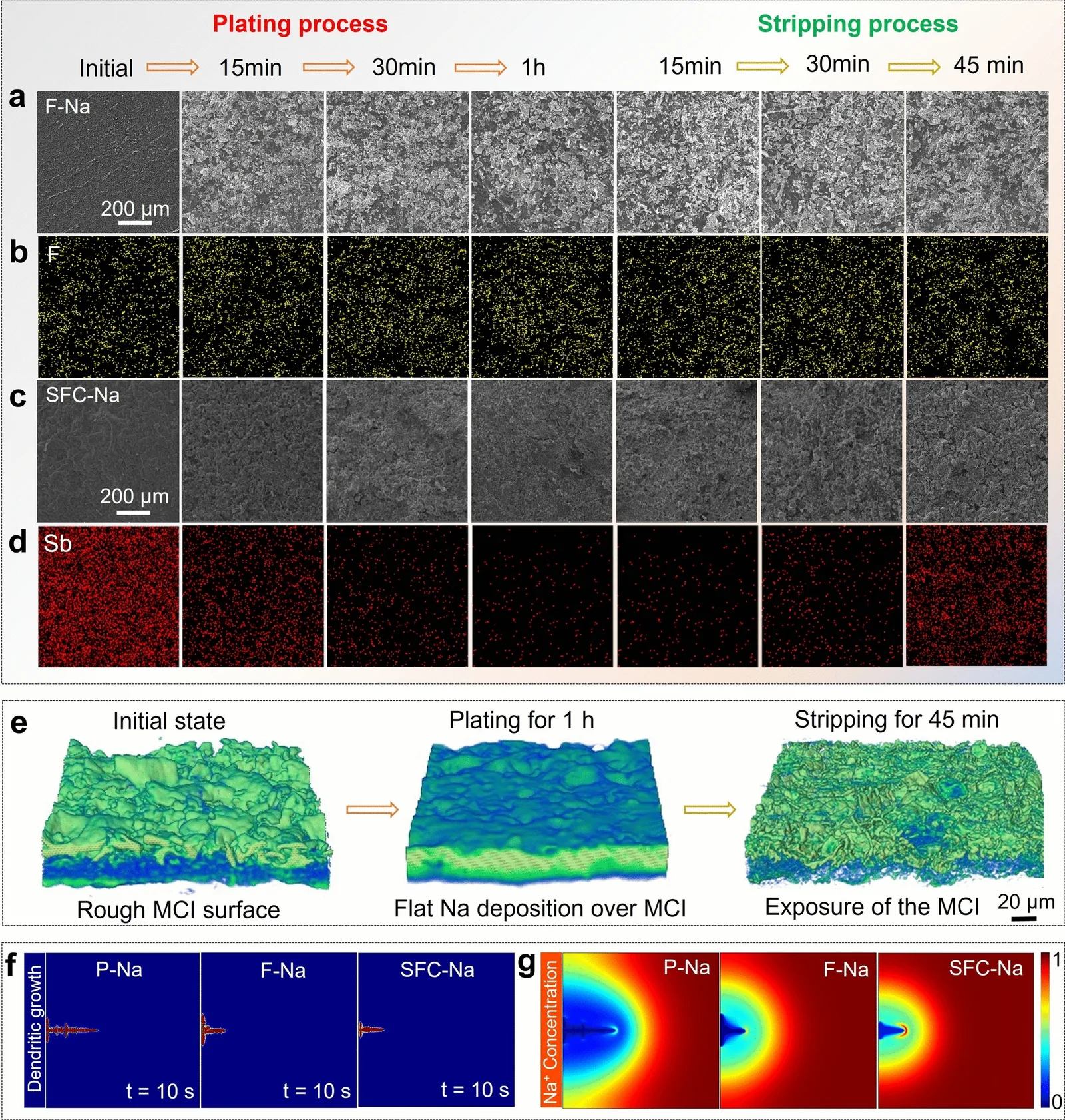
Na plating/stripping behavior. **a, b** SEM images and corresponding F element mapping of the F-Na anode during the Na plating/stripping process. **c, d** SEM images and corresponding Sb element mapping of SFC-Na anode during Na plating/stripping process. **e** 3D morphology of SFC-Na under different Na plating/stripping times. Phase-field simulation of **f** Na dendrite growth and **g** Na^+^ concentration near the artificial layer region of anodes

The properties of the MCI and the specific Na plating/stripping behavior are further studied. Symmetric cells are subjected to galvanostatic cycling to examine the microscopic morphology of deposited Na at various stages, with selected samples characterized by SEM. As shown in Fig. 4a, b, the persistent presence of the F signal throughout cycling indicates that Na⁺ plating and stripping occur through the ICI. Repeated Na⁺ penetration can damage the ICI, triggering Na dendrite growth and interfacial side reactions. In contrast, for the MCI (Fig. 4c, d), the Sb signal becomes obscured during Na deposition and sharpens upon Na stripping, suggesting that Na plating/stripping occurs on the MCI surface. Moreover, the deposited Na layer is readily removed during stripping, demonstrating the high electrochemical reversibility of the SFC-Na anode. This special phenomenon also confirms that the introduction of CMC binder does not affect the electronic conductivity of MCI layer. To further visually observe the Na plating/stripping behavior on the surface of SFC-Na, the synchrotron-based micrometer-level X-ray imaging is performed [41]. As shown in Fig. 4e, the initial rough surface displays the microstructure of MCI corresponding to the SEM images in Fig. 1f. As the Na deposition process progresses, the rough surface becomes flat due to being covered by Na metal. Furthermore, the reappearance of MCI after 45-min stripping confirms the surface-induced “top” Na deposition mechanism on the surface of SFC-Na.

Phase-field simulations further substantiate the superiority of the MCI. Based on the data from Fig. S17, the Na⁺ diffusion coefficient of the SEI layer is calculated for various anodes. As shown in Fig. S23, the construction of the MCI enhances the Na⁺ diffusion coefficient, which contributes to mitigating the concentration gradient across the corresponding SEI layer and promotes a more uniform distribution for the migration and detachment of unreacted Na⁺. To gain deeper insights into the mechanisms of Na dendrite growth, an electrochemical-mechanical phase-field model is established. Snapshots of dendrite evolution on P-Na, F-Na, and SFC-Na anodes reveal distinct growth patterns (Fig. 4f, g). In the absence of a sodiophilic layer, the P-Na anode exhibits severe filamentary dendritic structures, where lateral branches emerge from the main arms and further evolve into new dendrites [14, 42]. Additionally, corresponding two-dimensional distributions of Na⁺ concentration and the local electric field (Fig. S24) show an accumulation of Na⁺ near the tip of protrusions, where an intensified local electric field accelerates dendritic growth [43]. In contrast, both F-Na and SFC-Na anodes form smaller dendrites with significantly suppressed growth rates, demonstrating the effective dendrite inhibition imparted by the artificial layer. Owing to the dual ion/electron conductivity, the MCI exhibits superior dendrite suppression capability.

### Interface Evolution

**Fig. 5:**
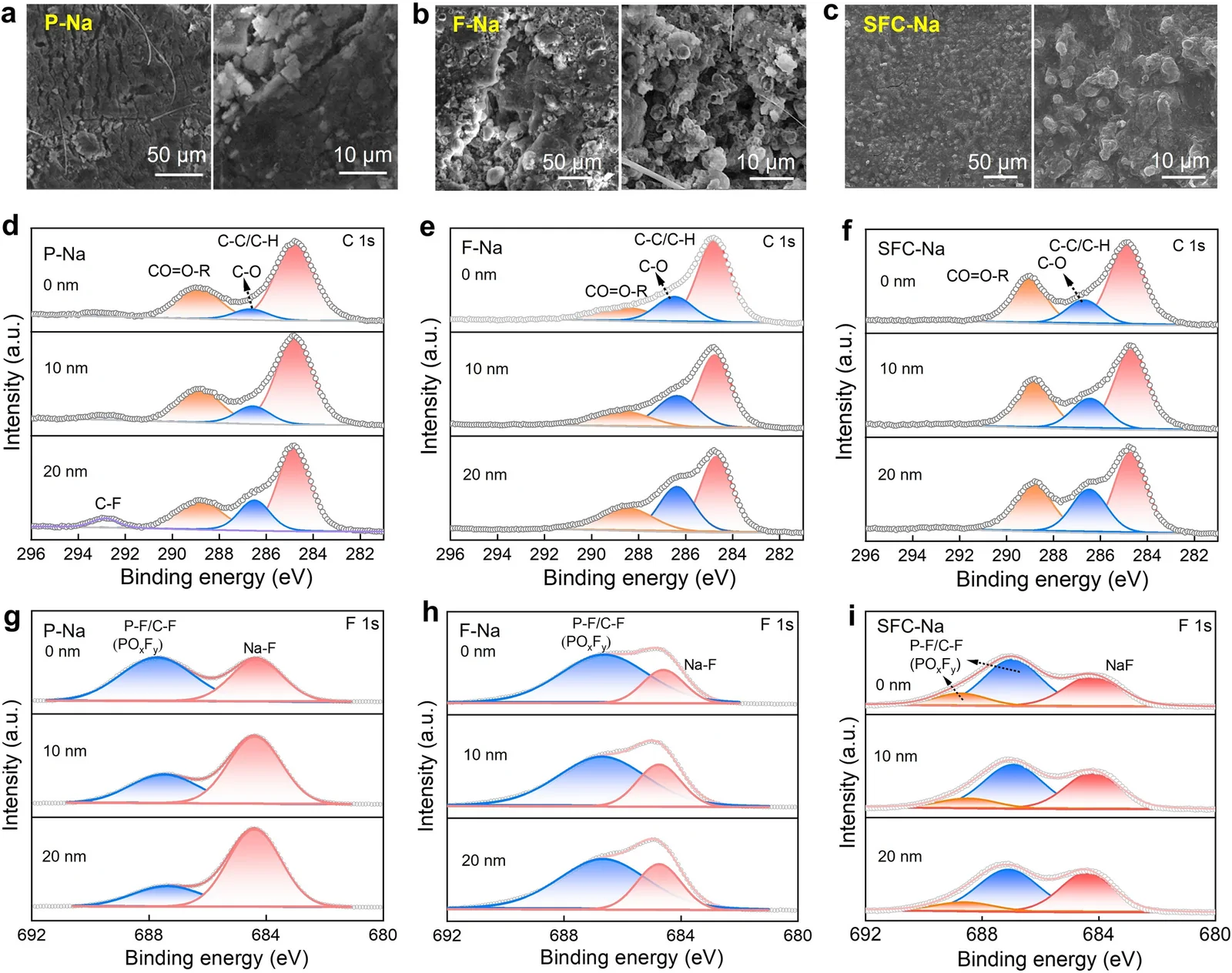
Microscopic morphology of the deposited Na and chemical components of the SEI layer. SEM images of **a** P-Na, **b** F-Na, and **c** SFC-Na anodes after 200 cycles in Na||Na symmetric cells at 0.5 mA cm^−2^. XPS spectra of C 1*s* of the SEI layer on **d** P-Na, **e** F-Na, and **f** SFC-Na surfaces. XPS spectra of F 1*s* of the SEI layer on the **g** P-Na, **h** F-Na, and **i** SFC-Na surfaces

To observe the surface micromorphology and chemical components of the F-Na and SFC-Na anodes, the symmetric cells are disassembled after cycling 200 cycles at 0.5 mA cm^−2^. SEM images (Fig. 5a–c) reveal severe dendrite growth on the F-Na anode, attributable to the inherent brittleness of the ICI and cyclic volume changes of the anode, which collectively cause progressive fracture of the sodiophilic layer and impaired electrochemical performance. In contrast, the MCI surface maintains a uniform and flat Na deposition layer, demonstrating the superior dendrite suppression capability afforded by its mixed ion and electron conductivity. Moreover, from the cross-sectional SEM images (Fig. S25), it can be observed that owing to the strong cross-linked network formed by CMC binder, the MCI layer still tightly adheres to the surface of Na metal substrate even after 200 cycles, preserving its structural integrity effectively. In addition, the morphology of Na metal after long-term deposition at 0.5 mA cm^−2^ is further observed. As shown in Fig. S26, The Na metal deposited on the surfaces of P-Na and F-Na show varying degrees of cracks and mossy Na deposition, while the Na metal deposited on the surface of SFC-Na is flat and dense, demonstrating the superiority of MCI in reducing Na nucleation barriers and promoting Na migration. Furthermore, the components of the SEI layer at different depths were performed. As shown in Fig. 5d–f, the SEI layer derived from the surface of the MCI has a more uniform C-containing composition in depth distribution, which is beneficial for the uniform charge transfer and Na^+^ diffusion [44]. Conversely, the chemical components of the SEI layer derived from the P-Na and F-Na surfaces vary greatly longitudinally, and continuous Na^+^ penetration can cause SEI rupture and dendrite growth (Fig. 5g–i). It is worth noting that the SEI layer derived from the MCI has a lower NaF component. NaF has a higher ion and electron diffusion energy barrier, which is not conducive to long-term rapid interfacial ion and electron transfer [45]. The ion–electron mixing conductivity of MCI changes the chemical components of the SEI layer, fundamentally improving the Na plating/stripping behavior at the interface.

### Electrochemical Performance of QSMBs

To evaluate the practical application of the SFC-Na anode, the QSMBs are assembled using an Na_3_V_2_(PO_4_)_3_ (NVP) cathode and DGPE. As shown in Fig. S27, SEM images and elemental mapping of the pristine NVP cathode reveal a granular morphology with uniformly distributed F and V elements, indicating a homogeneous distribution of the active material. Furthermore, after incorporating the DGPE, the enhanced F signal confirms that the electrolyte containing fluoroethylene carbonate has penetrated the bulk of the NVP cathode.

**Fig. 6:**
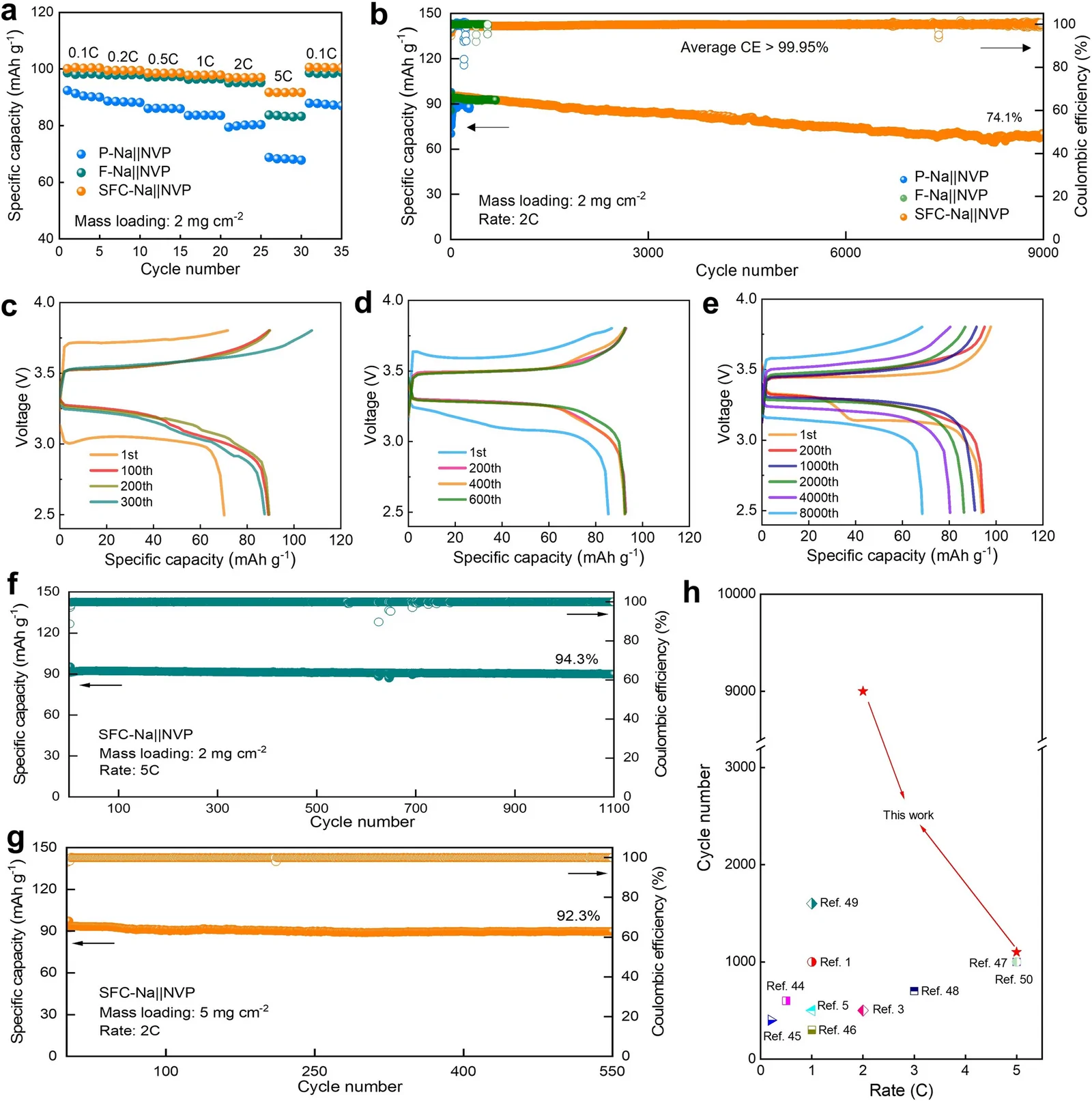
Electrochemical performance of QSMBs. **a** The rate capability of QSMBs with various anodes. **b** Long-cycling at 2C and corresponding charging/discharging curves with **c** P-Na, **d** F-Na, and **e** SFC-Na anodes. **f** Long-cycling of QSMBs with SFC-Na anode at 5C. **g** Long-cycling of QSMBs with 5 mg cm^−2^ mass loading of NVP cathode material. **h** Comparison of cycling life and rate performance of the QSMBs with the SFC-Na anode with the reported works

The rate capability of QSMBs using P-Na, F-Na, and SFC-Na anodes is compared in Fig. 6a. The cells with SFC-Na and F-Na anodes deliver similar specific capacities at low rates, both exceeding that of the cell with the P-Na anode. As the rate increases, the cell with SFC-Na achieves the highest capacity of 91.8 mAh g^−1^ at 5C, outperforming the cell with F-Na (83.8 mAh g^−1^). Furthermore, the long-cycling performance of QSMBs is evaluated at room temperature. As shown in Fig. 6b, the QSMBs with SFC-Na anode allow a remarkable specific capacity of 67.9 mAh g^−1^ at 2C after 9000 cycles, corresponding to an excellent capacity retention of 74.1%. Moreover, the average CE over 9000 cycles is greater than 99.95%, demonstrating improved Na plating/stripping behavior. In contrast, cells with P-Na and F-Na anodes fail after only about 250 and 680 cycles along with fluctuating Coulomb efficiency, respectively. Notably, as shown in the charging/discharging curves (Fig. 6c–e), the QSMBs with SFC-Na anode have the lowest polarization, which is owing to the artificial sodiophilic layer with strong adhesion force for suppressing interfacial side reactions and Na dendrite growth. Especially, the dual plateau of the initial discharging curve of the cells with SFC-Na anode originates from the overpotential required for Na at the bottom to pass through the MCI layer, demonstrating the bottom Na source compensation during discharging process. After multiple cycles, the dual platform disappears, indicating the formation of a stable SEI layer on the surface of the Na deposition layer.

To further highlight the potential of the practical application of the SFC-Na anode, long-cycling performance is conducted under high rate and mass loading. As shown in Fig. 6f, the QSMBs with SFC-Na anode exhibit outstanding cycling stability with an excellent capacity retention of 94.3% after 1100 cycles at 5C and a high specific capacity (89.96 mAh g^−1^). Additionally, even at a high mass loading of 5 mg cm^−2^, the QSMBs with the SFC-Na anode still exhibit satisfactory cycling stability for 550 cycles at 2C with no significant decay (Fig. 6g). Surprisingly, the performance of cells with the SFC-Na anode is superior to previously reported QSMBs (Fig. 6h and TableS3) [1, 3, 5, 46–52] and the cells with other alloy anodes (Table S4 [53–58], demonstrating the superiority of the surface-induced “top” Na deposition mechanism. Moreover, a 200 mAh-class pouch cell based on SFC-Na anode and high mass loading NVP cathode (20 mg cm^−2^) exhibits favorable cycling stability without obvious capacity degradation after 40 cycles at 1C (Fig. S28), highlighting the potential for large-scale application of this strategy. These findings suggest that implementing the SFC-Na anode can substantially enhance the cycling stability of QSMBs under diverse conditions.

## Conclusions

In summary, we propose a surface-induced “top” Na deposition paradigm enabled by an artificial interphase layer composed of NaF and Na_3_Sb, constructed via a simple chemical soaking method. Driven by the electron conductivity of Na_3_Sb, Na directly deposits on the surface of the MCI instead of crossing through the MCI, avoiding physical damage to the MCI and poor interface contact. Furthermore, the composite of NaF and Na_3_Sb accelerates interfacial Na^+^ diffusion and suppresses interface side reactions, contributing to long-term stable Na plating/stripping. In addition, the induced dense Na deposition layer promotes the formation of the stable and durable SEI layer, which is beneficial for the long-term stability of GPEs/anode interface. As a result, the QSMBs equipped the optimized SFC-Na anode, NVP cathode, and DGPE exhibit significantly enhanced cycling stability and rate capability. This work provides a unique perspective for designing an artificial interphase layer and regulating Na plating/stripping behavior, paving the way for the development of practical QSMBs.

## Supplementary Information

Below is the link to the electronic supplementary material.

Supplementary file1 (DOCX 6860 KB)
