# Supplementary material for: Ultra-Sodiophilic Mixed Conductor Interphase Enabling Uniform Top Deposition for Quasi-Solid-State Sodium-Metal Batteries
Source: Nanomicro Lett. 2026 Jun 29;18:417. doi: 10.1007/s40820-026-02256-y (PMC13315381; doi:10.1007/s40820-026-02256-y)
Supplement: Supplementary file 1 — Supplementary file1 (DOCX 6860 KB) [file 40820_2026_2256_MOESM1_ESM.docx]

Supporting Information for

**Ultra-Sodiophilic Mixed Conductor Interphase Enabling Uniform Top Deposition for Quasi-Solid-State Sodium Metal Batteries**

Chunching Lu^1, #^, Guangxiang Zhang^1, #^, Yuxiang Niu^2^, Yupeng Zhu^2^, Siyuan Li^2^, Hua Huo^1^, Yulin Ma^1,^ *, Pengjian Zuo^1^, Geping Yin^1^, Yunzhi Gao^1^, Liguang Wang^3,^ *, Chuankai Fu^1,^ *, and Wei Chen^2,^ *

^1^State Key Laboratory of Space Power-Sources, School of Chemistry and Chemical Engineering, Harbin Institute of Technology, Harbin 150001, P. R. China

^2^Department of Chemistry, National University of Singapore, 3 Science Drive 3, 117543, Singapore

^3^College of Chemical and Biological Engineering, Zhejiang University, Hangzhou 310058, P. R. China

^#^Chunching Lu and Guangxiang Zhang contributed equally to this work.

*Corresponding authors. E-mail: phycw@nus.edu.sg (Wei Chen); chuankaifu@hit.edu.cn (Chuankai Fu); mayulin@hit.edu.cn (Yulin Ma); wanglg@zju.edu.cn (Liguang Wang)

**Supplementary Figures and Tables**

**
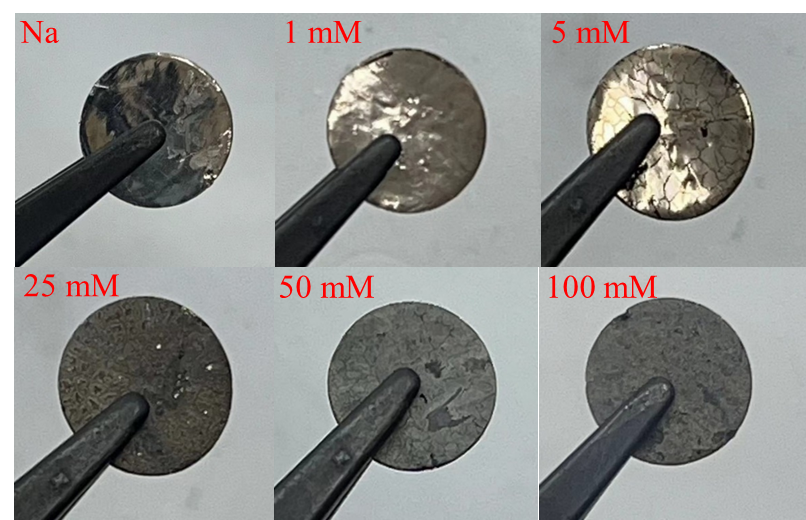
**

**Fig. S1** Optical photos of the Na anode surface after treatment with different concentrations of SbF_3_ in DME.

Optical photos indicate that the protective layer on the Na metal surface is most compact when the concentration of SbF_3_ is 100 mM.

**Fig. S2** Cycling performance of Na||Na symmetric cells with Na anode after chemical treatment in different SbF_3_ concentrations at 0.1 mA cm^−2^.

The cycling stability of Na metal treated with 100 mM SbF_3_ solution is optimal.


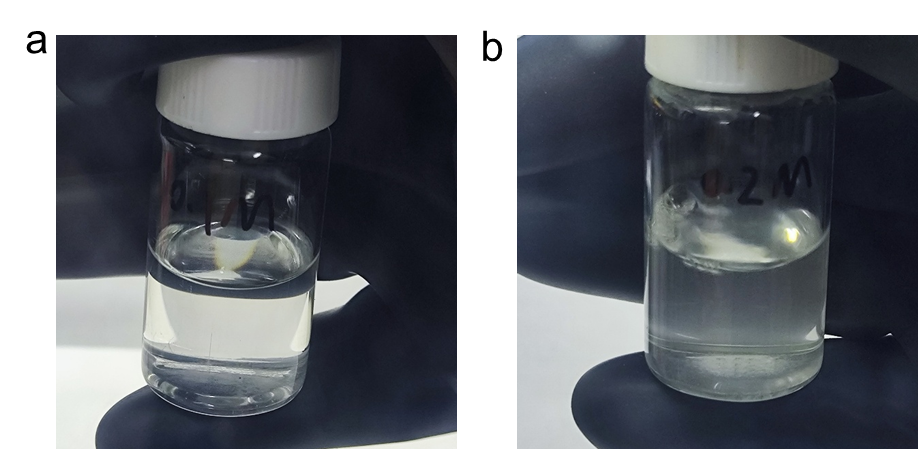


**Fig. S3** Optical photos of **a** 0.1 M and **b** 0.2 M SbF_3_ in DME.

The solution of 0.2 M SbF_3_ in DME shows a turbid state, indicating that SbF_3_ cannot be completely dissolved.

**
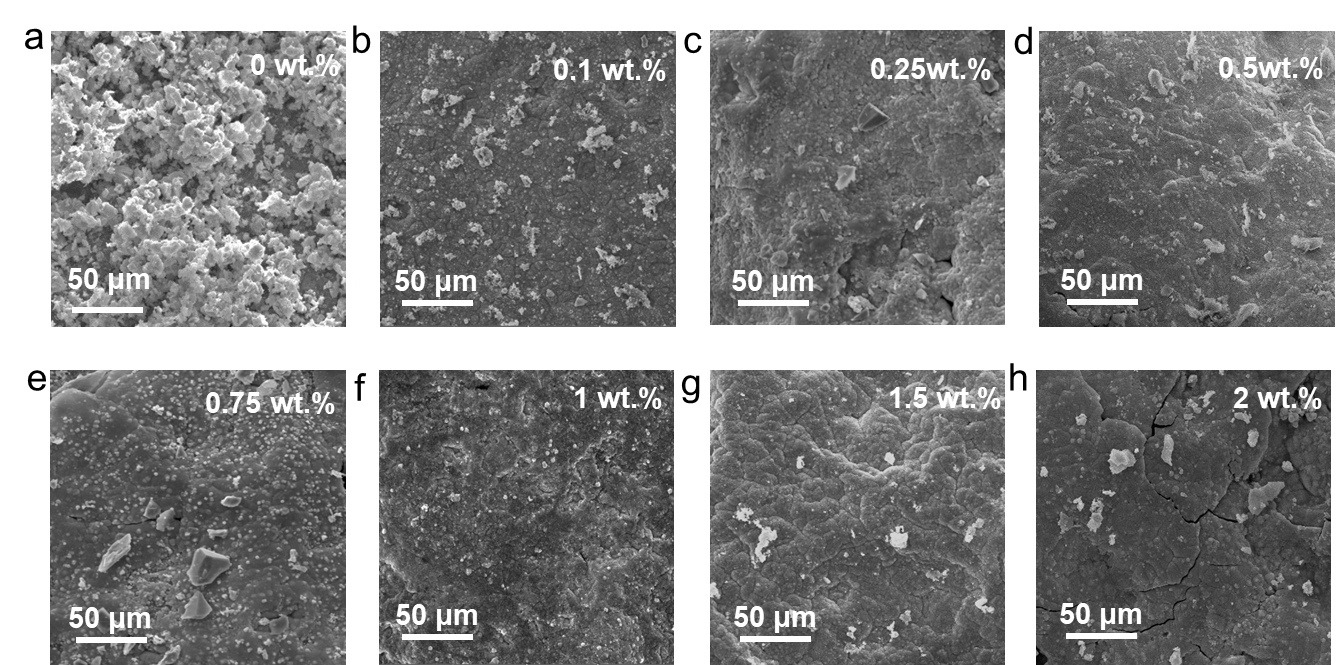
**

**Fig. S4** The SEM images of the SFC-Na anode after treatment by SbF_3_/DME solutions with different CMC concentrations.

When the CMC content in SbF_3_/DME solution is 1 wt.%, the MCI layer is the densest and uniform.


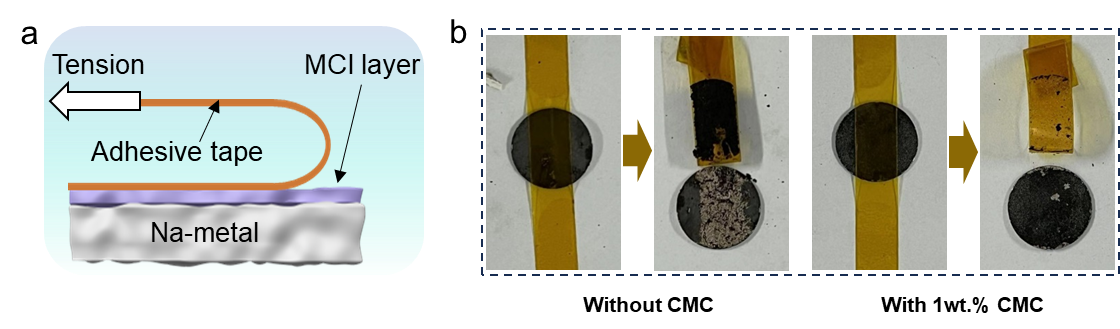


**Fig. S5 a** Schematic diagram of adhesion experiment, **b** adhesion experiment of MCI layer without or with 1 wt.% CMC.

Adhesion tests show that the addition of 1 wt.% CMC effectively promotes the adhesion between the MCI layer and the Na-metal substrate.

**Fig. S6** XPS spectra of C 1s on the SFC-Na surface.

C=O-OR, C-O, and other organic components generated by DME solvent can improve the flexibility of the MCI layer.

**
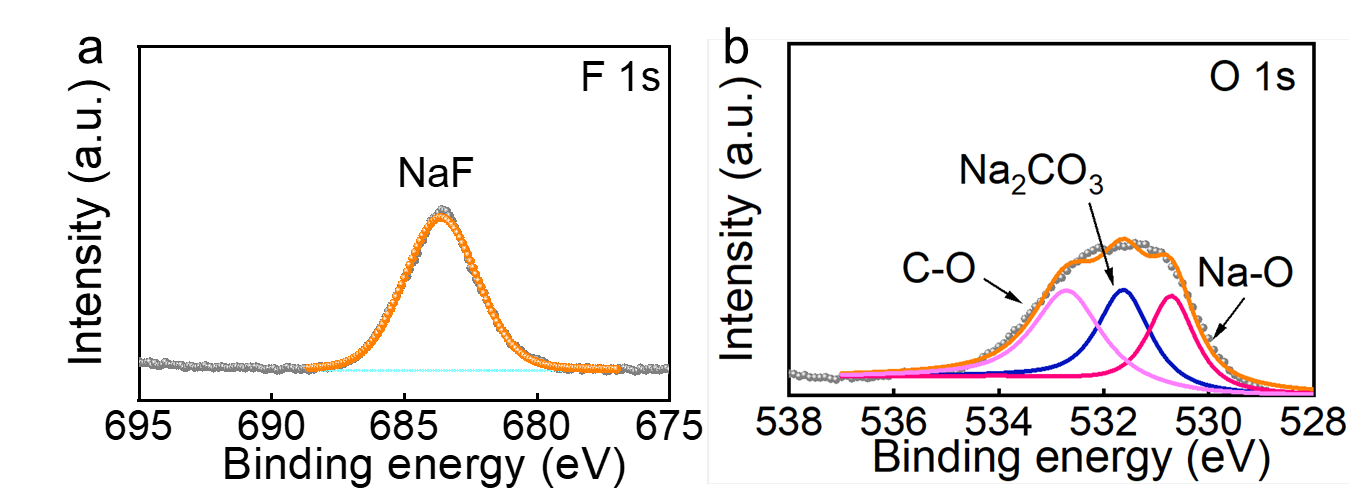
**

**Fig. S7** XPS spectra of **a** F 1s and **b** O 1s on the F-Na surface.

Organic components and NaF are also detected on the surface of F-Na.

**
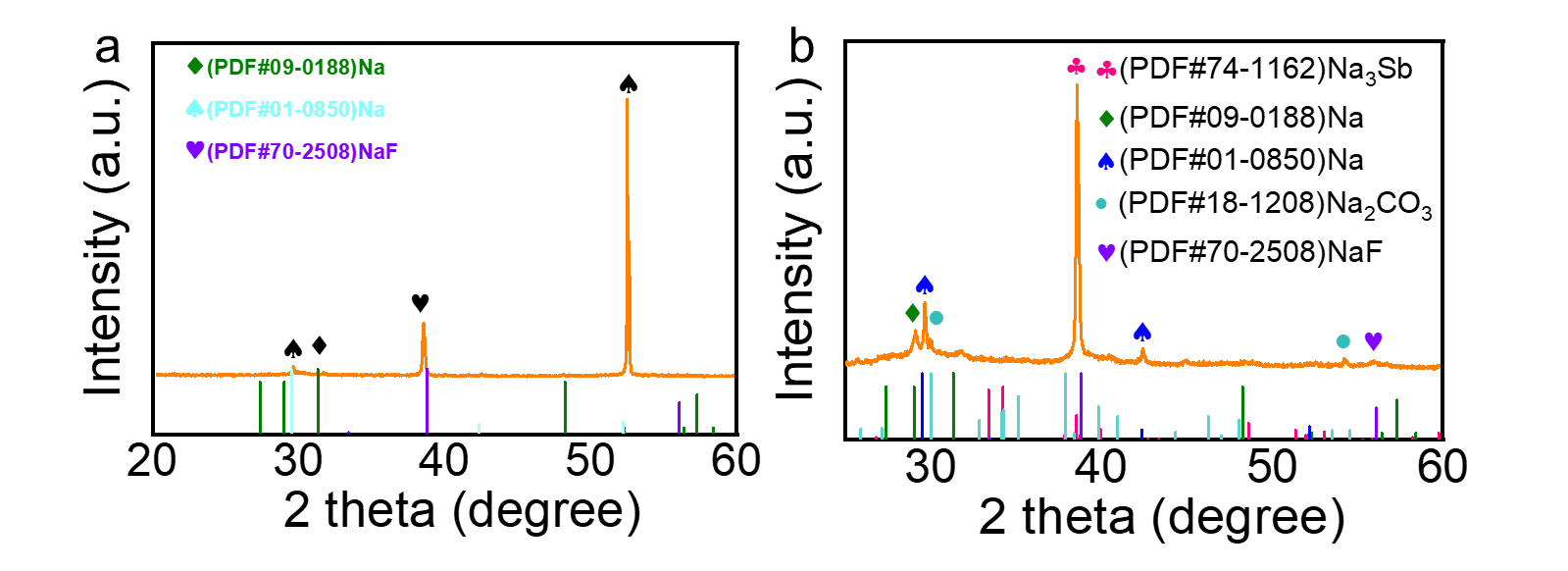
**

**Fig. S8** XRD spectra of **a** F-Na and **b** SCF-Na.

XRD analysis further verifies the phase composition, with distinct diffraction peaks corresponding to NaF (PDF#70-2508) and Na₃Sb alloy (PDF#74-1162) in the surfaces of F-Na and SFC-Na anodes.

**
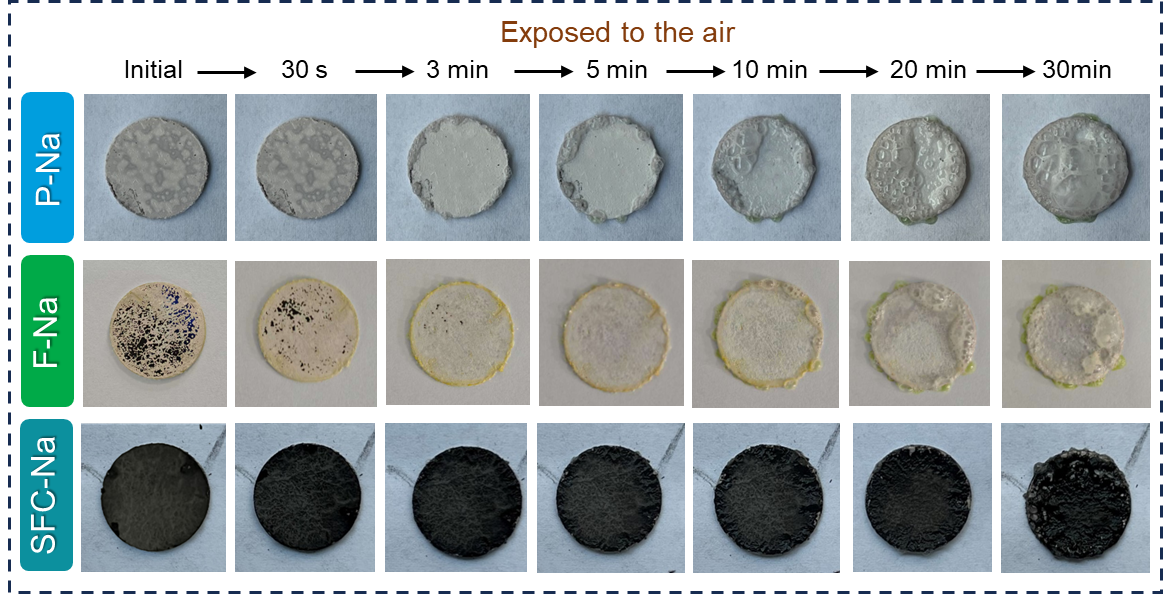
**

**Fig. S9** The air stability of P-Na, F-Na, and SCF-Na.

Owing to the high compactness and excellent chemical stability of MCI, the SFC-Na anode can remain stable for 30 min in the air, which is superior to that of F-Na (3 min) and P-Na (5 min).

**
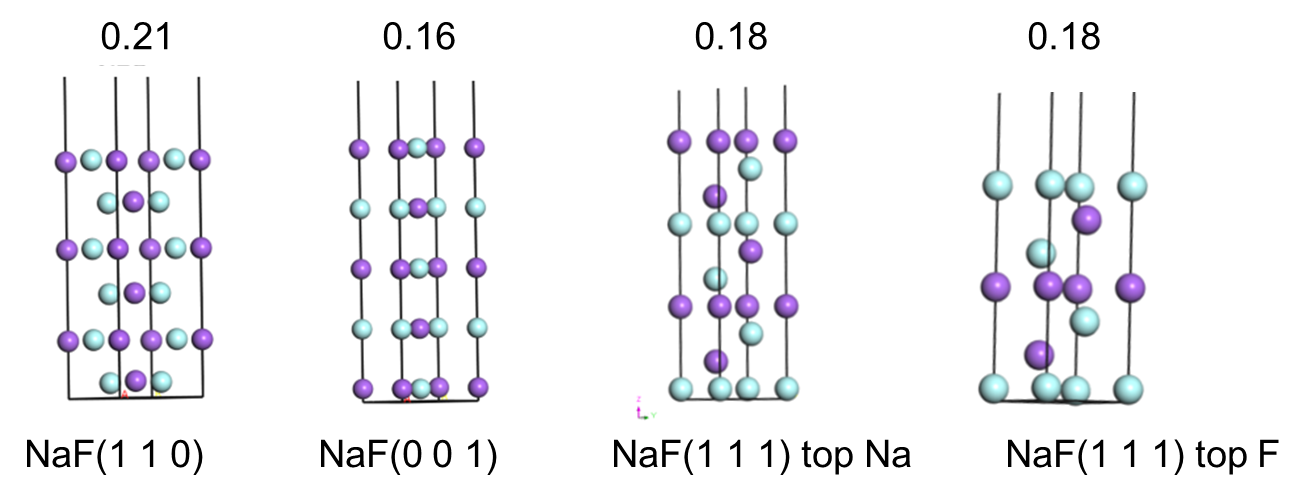
**

**Fig. S10** Surface energies (eV/Å^2^) of NaF on the surface of SFC-Na.

The evolution mechanism of Na deposition on the MCI is elucidated by DFT, employing modeled NaF (001) surfaces with the lowest surface energies.

**
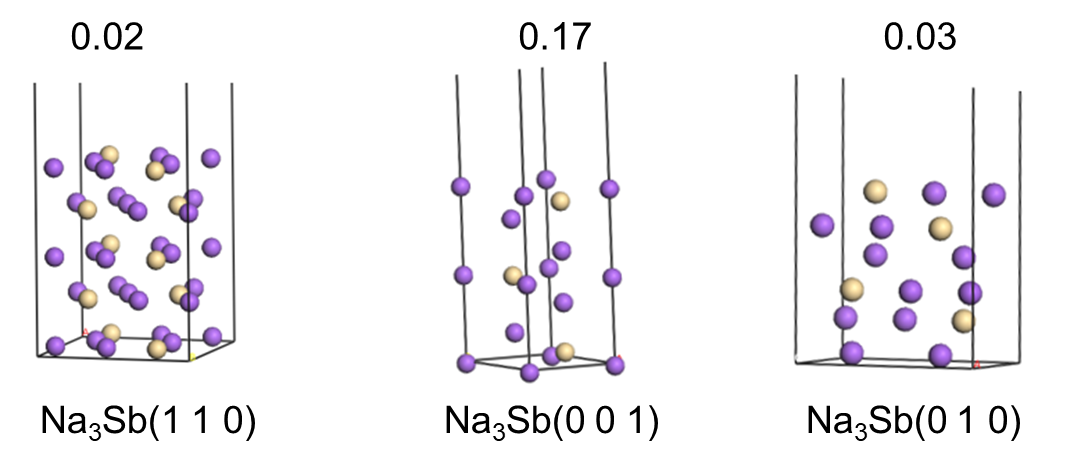
**

**Fig. S11** Surface energies (eV/Å^2^) of Na_3_Sb on the surface of SFC-Na.

The evolution mechanism of Na deposition on the MCI is elucidated by DFT, employing modeled Na_3_Sb (110) surfaces with the lowest surface energies.


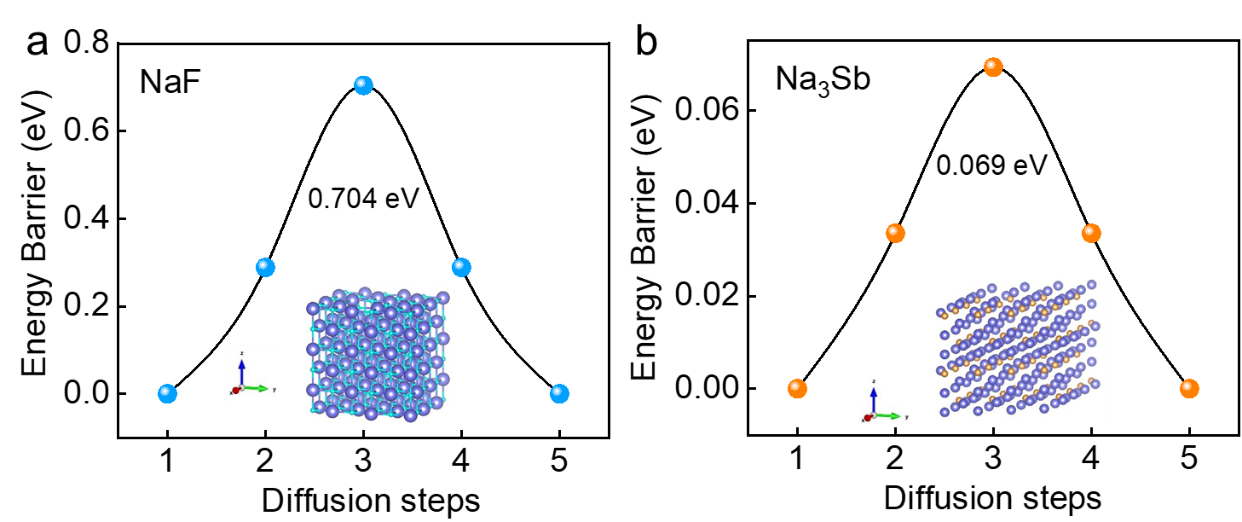


**Fig. S12** DFT simulations of Na^+^ diffusion in NaF **a** and Na atom diffusion in Na_3_Sb **b**.

The Na^+^ diffusion energy barrier in NaF is much higher than Na atom diffusion energy barrier in Na_3_Sb, demonstrating the significant role of Na_3_Sb in promoting electrochemical kinetics.


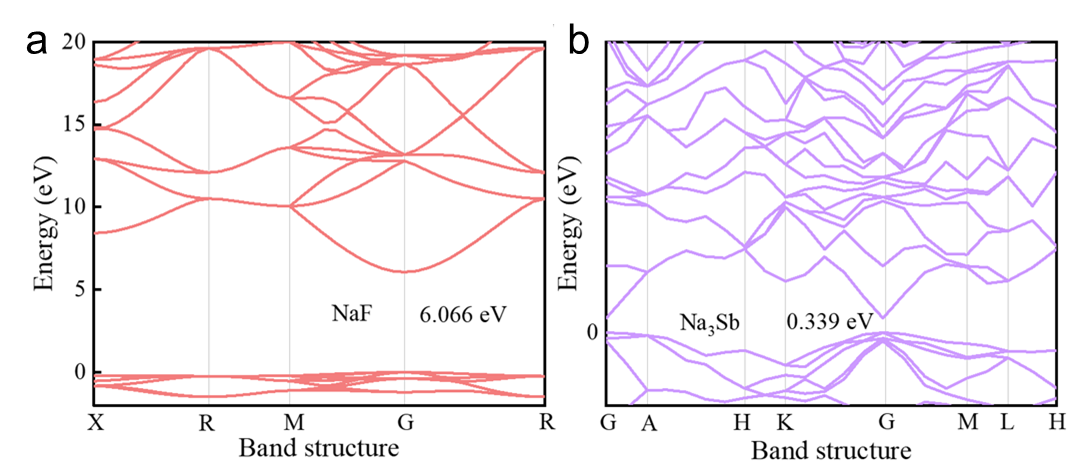


**Fig. S13** The band gap of **a** NaF and **b** Na_3_Sb.

NaF in MCL exhibits high electron blocking ability, which can effectively prevent electron tunneling, while Na_3_Sb phase can improve electron conductivity, achieve rapid electron transfer, and avoid charge accumulation.

**
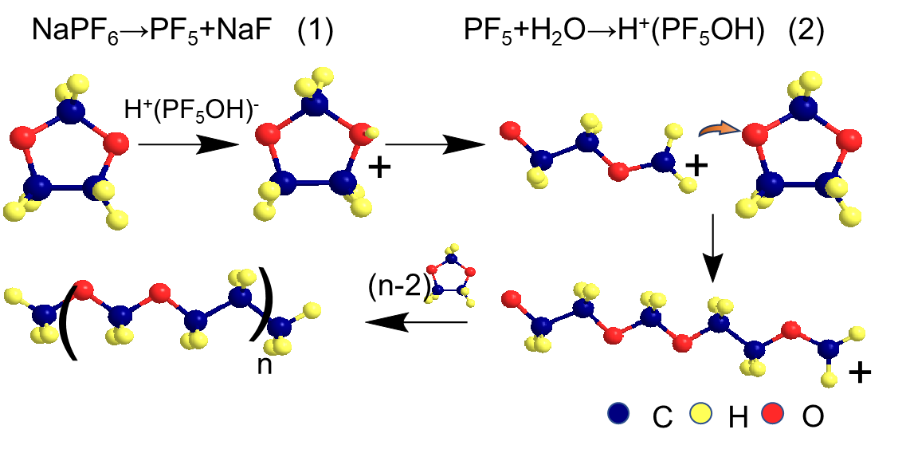
**

**Fig. S14** Ring-opening polymerization mechanism of DOL.

The polymerization mechanism involves a Lewis acid PF_5_^–^ species derived from NaPF_6_, which reacts with trace water to generate protons that initiate the ring-opening polymerization of DOL.

**
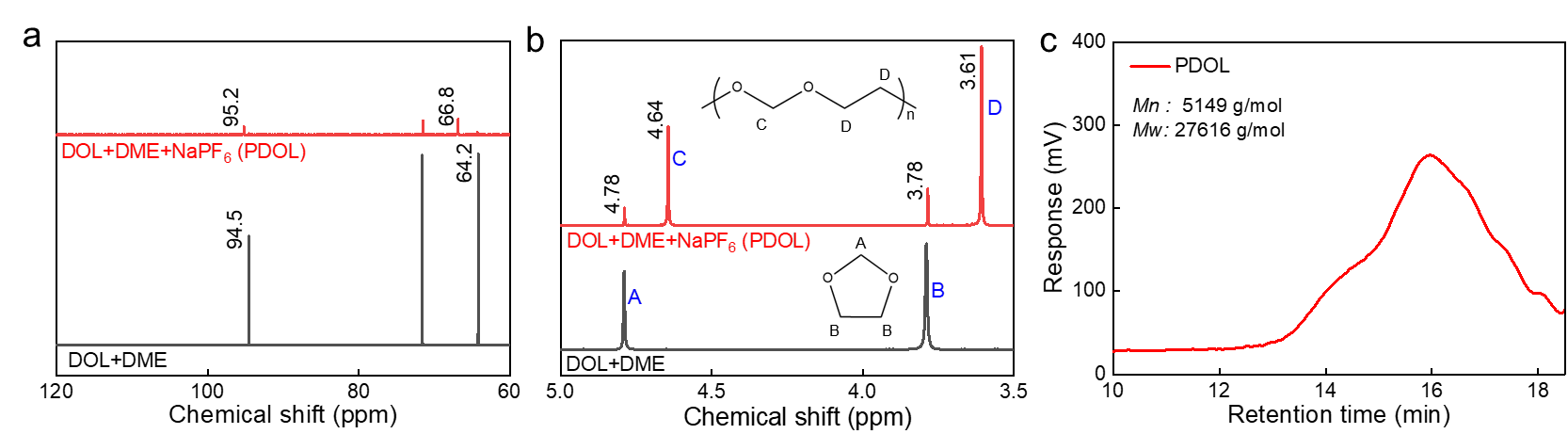
**

**Fig. S15 a** ^13^C NMR and **b** ^1^H NMR spectra before and after DOL polymerization. **c** GPC spectrum of DGPE.

DOL was polymerized successfully, achieving a polymerization degree of 89.7% and number-average and weight-average molecular weight of 5,149 g mol^-1^ and 27,616 g mol^-1^, respectively.

**Fig. S16** The impedance of Na||Na symmetric cells under different storage times before cycling.

After long-term storage, the impedance of Na||Na symmetric cells remain almost unchanged, proving the good compatibility between DGPE and SFC-Na.

**
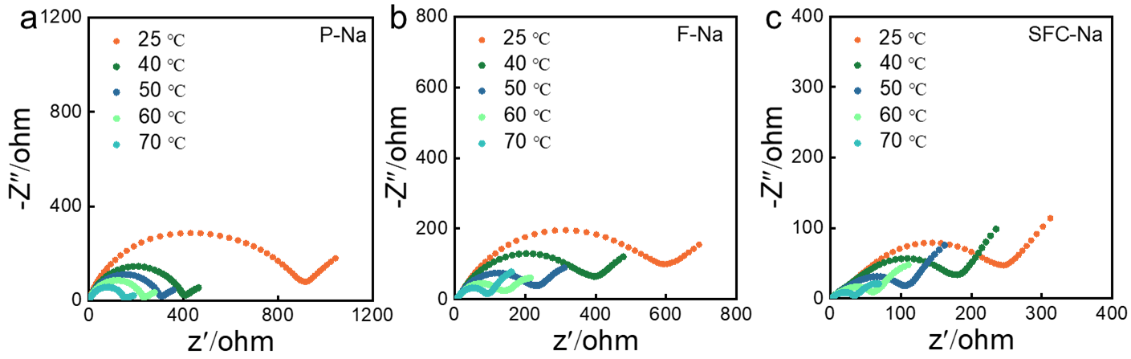
**

**Fig. S17** Nyquist plots of **a** P-Na, **b** F-Na, and **c** SFC-Na in the Na||Na symmetric cells at different temperatures after 5 cycles.

The impedances of Na^+^ transport across the SEI (R_SEI_) layer and charge-transfer (R_ct_) at different temperatures are further measured by EIS to reflect the superiority of the MCI in inducing the construction of the stable and robust SEI layer and interfacial Na^+^ migration.

**
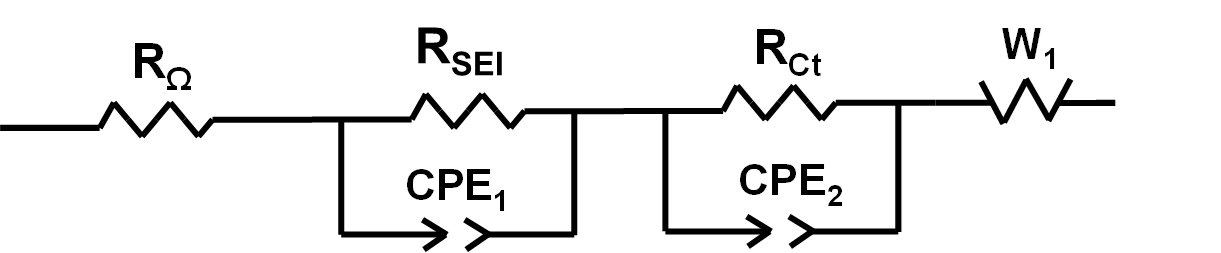
**

**Fig. S18** Equivalent circuit of impedance fitting.

The EIS values are fitted by the corresponding equivalent circuit.

**
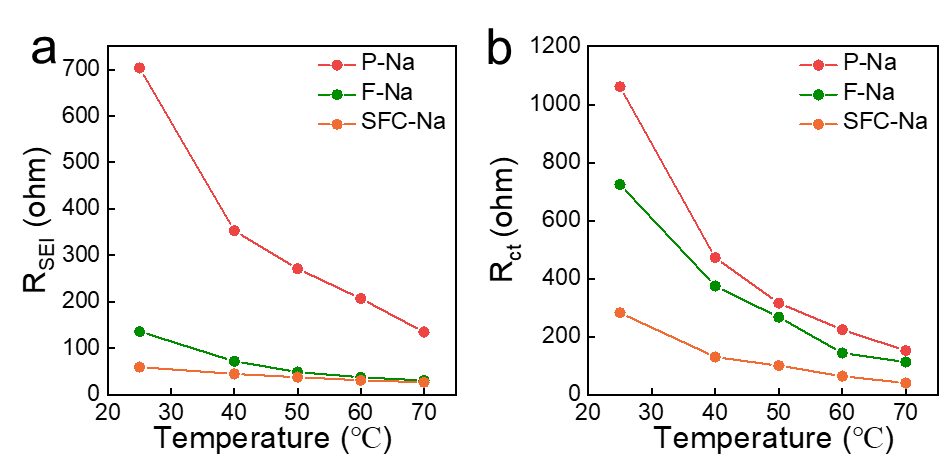
**

**Fig. S19** Impedance values of **a** R_SEI_ and **b** R_ct_.

The R_SEI_ and R_ct_ of quasi-solid-state symmetrical cells with SFC-Na anodes are markedly lower than those of cells with P-Na or F-Na anodes, demonstrating clear superiority.

**Fig. S20** Galvanostatic long-term discharging performance of Na||Na symmetric cells with P-Na, F-Na, and SFC-Na anodes.

The cells with P-Na and SF-Na stabilized for only 2 h and 8 h, respectively, before short circuit, whereas the SFC-Na cell maintained a stable voltage plateau at 80 mV for 22 h, demonstrating effective suppression of Na dendrite growth.


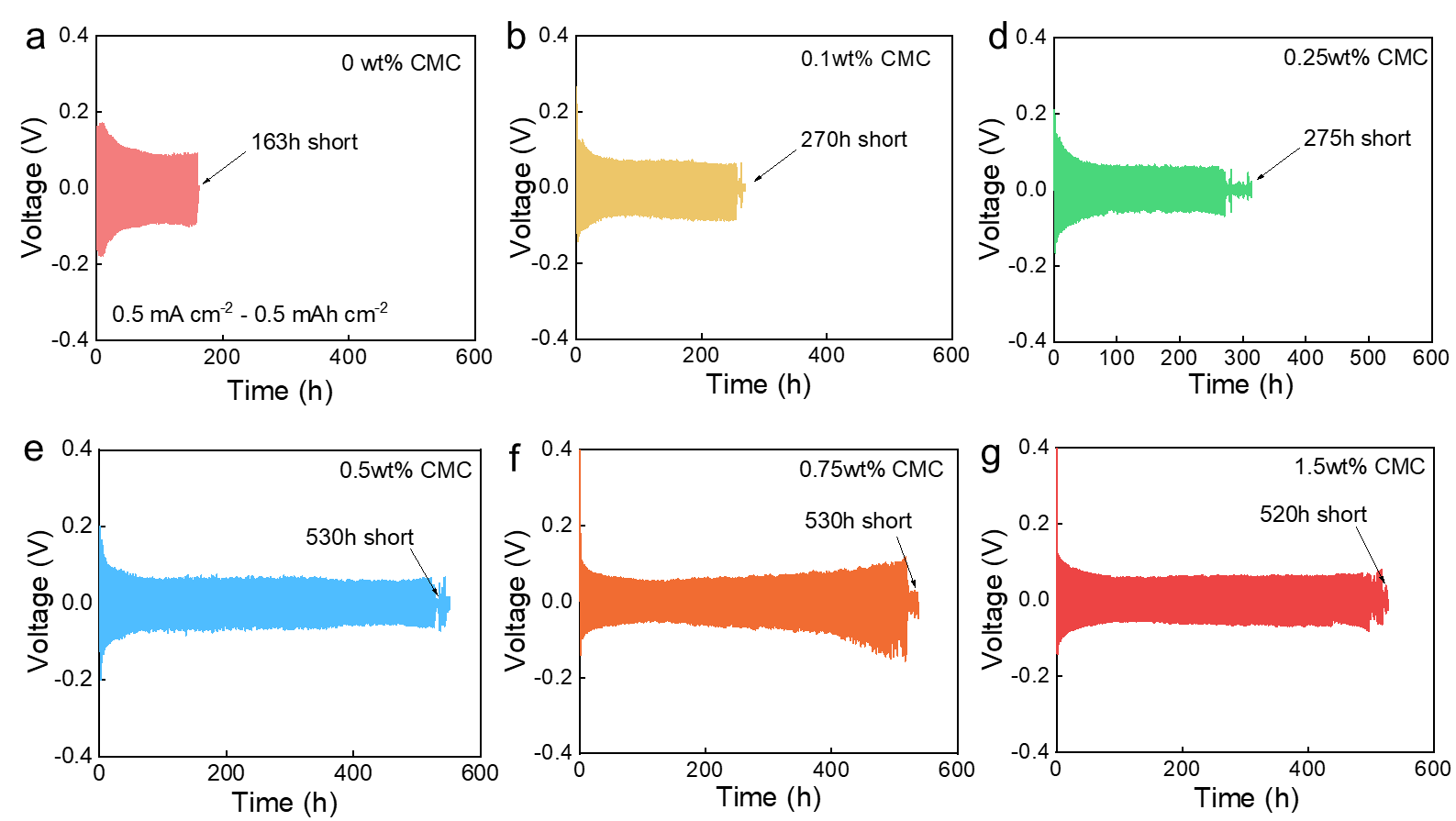


**Fig. S21** Cycling performance of Na||Na symmetric cells with SFC-Na anode with different CMC contents at 0.5 mA cm^−2^.

The Na||Na symmetric cells with an SFC-Na anode consisting of 1wt.% CMC exhibits the best cycling stability.

**Fig. S22** Cycling performance of Na||Na symmetric cells with SFC-Na anode at 2.0 mA cm^−2^.

Even at a higher current density of 2 mA cm⁻^2^, the SFC-Na cells can stably cycle for 180 h without short circuit.

**Fig. S23** Correlation between Z-real and ω^-1/2^ for P-Na, F-Na and SFC-Na anodes.

The construction of the MCI enhances the Na⁺ diffusion coefficient, thereby mitigating the concentration gradient across the corresponding SEI layer and promoting a more uniform distribution of Na⁺ during migration and stripping.

**
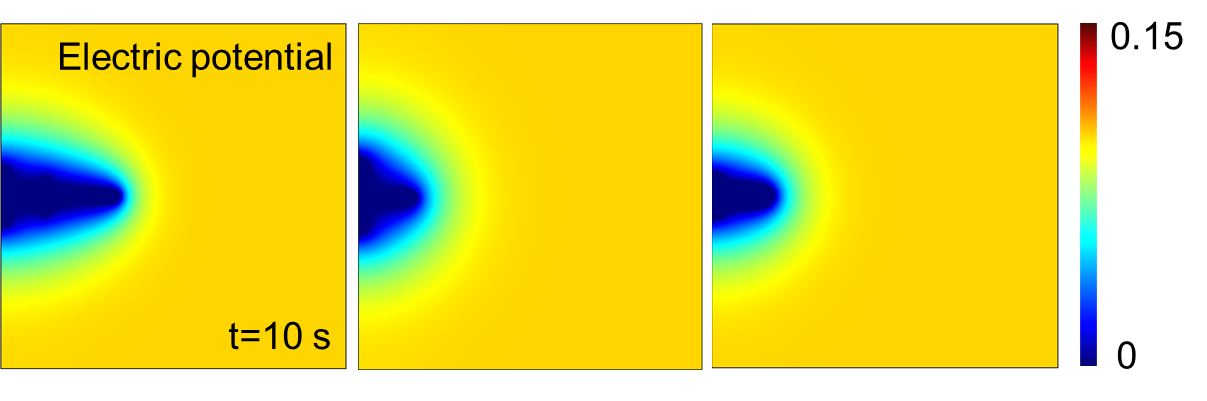
**

**Fig. S24** The images of **a** P-Na, **b** F-Na, and **c** SFC-Na show the 2D distribution of the local electric field at t = 10 s.

The corresponding two-dimensional distributions of Na⁺ concentration and the local electric field reveal Na^+^ accumulation near the protrusion tip, where an intensified local electric field further accelerates dendritic growth.


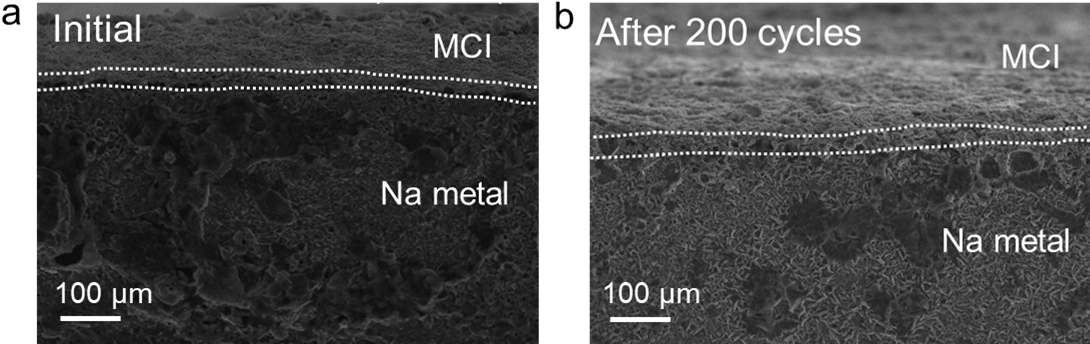


**Fig. S25** Cross-sectional SEM images of SFC-Na anode: **a** initial state and **b** after 200 cycles.

After long-term cycling, the MCI layer remains firmly adhered to the Na metal surface.


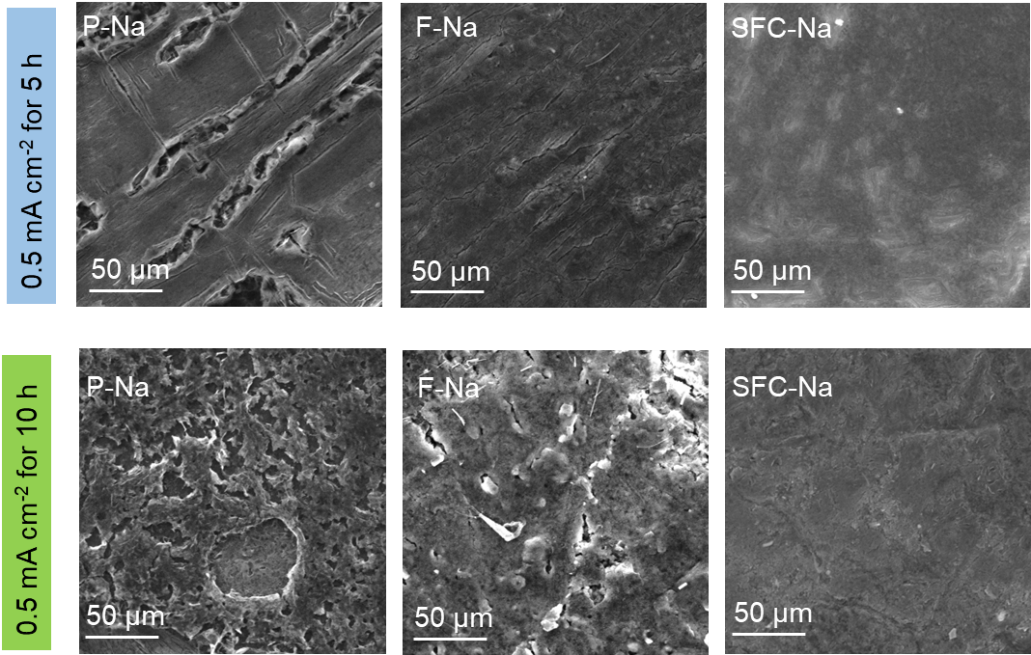


**Fig. S26** The SME images of the deposited Na on the P-Na, F-Na, and SFC-Na surfaces at 0.5 mA cm^-2^ for 5 h and 10 h.

Driven by the strong Na^+^ affinity of SFC-Na, the Na metal deposited on its surface exhibits a smoother morphology.

**
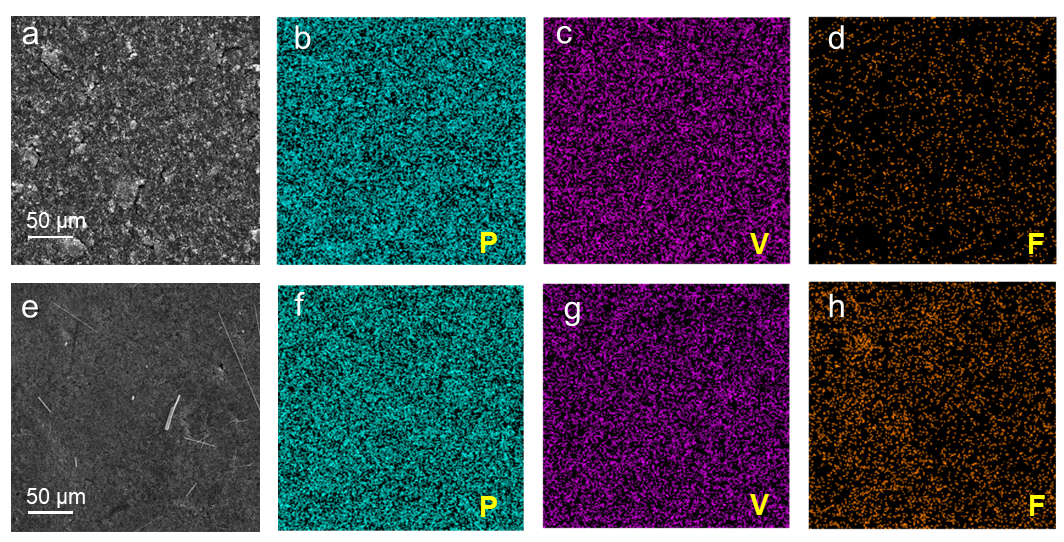
**

**Fig. S27** Comparison of the morphology of Na_3_V_2_(PO_4_)_3_ (NVP) cathode before **a** and after gelation **e**, with corresponding surface EDX mappings shown in **b**-**d**, **f**-**h**, respectively.

SEM images and elemental mappings of the pristine NVP cathode reveal a granular morphology with uniformly distributed F and V elements, indicating a homogeneous distribution of the active material and electrolyte.

**
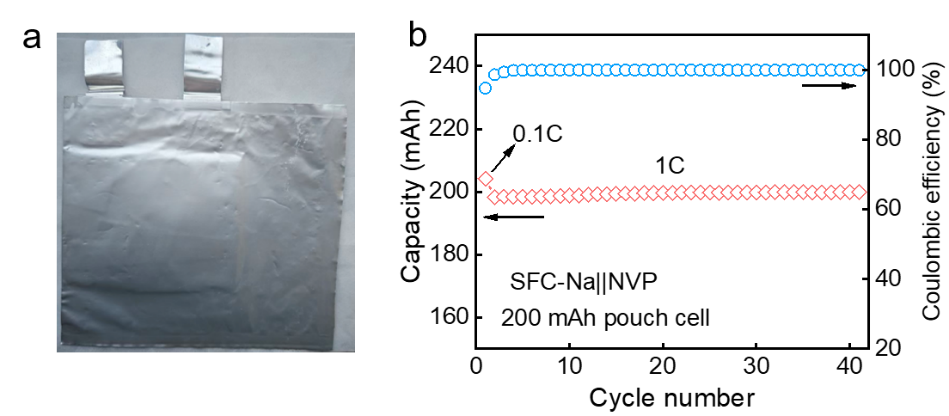
**

**Fig. S28** Optical photo **a** and cycling performance **b** of SFC-Na||NVP pouch cell with a capacity of ~200 mAh.

The 200 mAh pouch cell based on SFC-Na anode and NVP cathode delivers stable capacity retention and high Coulombic efficiency over 40 cycles at 1C.

**Table S1** Surface energy (eV Å^-2^) of different crystal surfaces of NaF and Na_3_Sb

| NaF | | Na_3_Sb | |
| --- | --- | --- | --- |
| Surface | Surface energy (eV) | Surface | Surface energy (eV) |
| (001) | 0.016 | (001) | 0.052 |
| (001) | 0.037 | (110) | 0.021 |
| (111) top-F | 0.186 | (010) | 0.033 |
| (111) top-Na | 0.186 |  |  |

**Table S2** Comparison of the lifespan of the SFC-Na anode with the reported works

| Artificial interphase | Electrolyte | Test conditions | Cycle life (hours) | Refs. |
| --- | --- | --- | --- | --- |
| Al_2_O_3_-Na | 1 M NaClO_4_-EC/DEC | 0.5 mA cm^-2^/1 mAh cm^-2^ | 120 | [9] |
| Multilayer-MoS_2_-Na | 1 M NaClO_4_-EC/DEC | 0.5 mA cm^-2^/0.25 mAh cm^-2^ | 100 | [33] |
| NaBr-Na | 1 M NaPF_6_-EC/PC | 1 mA cm^-2^/1 mAh cm^-2^ | 250 | [34] |
| Na_x_Sn_y_-Na | 1 M NaPF_6_-EC/PC | 0.5 mA cm^-2^/0.25 mAh cm^-2^ | 1000 | [35] |
| Na_3_PS_4_-Na | 1M NaPF_6_-EC/PC | 1 mA cm^-2^/ 1 mAh cm^-2^ | 275 | [36] |
| Na_x_Sn_y_-Na | 1 M NaPF_6_-EC/DMC | 0.25 mA cm^-2^/0.125 mAh cm^-2^ | 600 | [37] |
| NaCl-Na | 1 M NaClO_4_-EC/DMC | 0.3 mA cm^-2^/ 0.3 mAh cm^-2^ | 1000 | [38] |
| ZMOF-NSC@Na | 1M NaFSI-EC/PC | 0.5 mA cm^-2^/ 0.5 mAh cm^-2^ | 900 | [39] |
| Na_3_P-Na | 1 M NaClO_4_- EC/DEC/FEC | 1 mA cm^-2^/1 mAh cm^-2^ | 500 | [40] |
| **SFC-Na** | **1M NaPF_6_- DOL/DME/FEC** | **0.5 mA cm^-2^/0.5 mAh cm^-2^** | **1000** | **This work** |

**Table S3** Surface energy (eV Å^-2^) of different crystal surfaces of NaF and Na_3_Sb

| Components of electrolytes | Test conditions | Cycle life | Capacity retention | Refs. |
| --- | --- | --- | --- | --- |
| DOL/Al(OTf)_3_/NaTFSI/FEC | NVP loading: 1.12 mg cm^-2^  Rate: 0.5C | 600 | 96.7% | [1] |
| TMPTA/NaPF_6_/EC/DEC  /FEC | NVP loading: 13.01 mg cm^-2^  Rate: 1C | 1000 | ~80% | [3] |
| PEGMA/TEP/NaTFSI/FEC/  AIBN | NVP loading: - mg cm^-2^  Rate: 0.2C | 400 | 91% | [5] |
| PEGMA/TEP/NaTFSI/  PVDF-HFP/FEC | NVP loading: 1.2 mg cm^-2^  Rate: 1C | 500 | 91.7% | [46] |
| BA/NaPF_6_/DEC/EC/AIBN | NVP loading: 1.5 mg cm^-2^  Rate: 1C | 300 | 83% | [47] |
| HMPP/ETPTA/NaClO_4_/  PC/FEC | NVP loading: 1 mg cm^-2^  Rate: 5C | 1000 | 97% | [48] |
| EA/EDA/NaPF_6_/SN/  PPO/FEC | NVP loading: 3 mg cm^-2^  Rate: 3C | 700 | 83.3% | [49] |
| PAN | NVP loading: 2.1mg cm^-2^  Rate: 1C | 1600 | 96.03% | [50] |
| PEGDA/PVDF-HFP/  NaTFSI/NaBOB/PC/EC | NVP loading: 1.8 mg cm^-2^  Rate: 2C | 500 | / | [51] |
| TMPTMA/HDDA/NaTFSI/  ABVN/FEC/DMC/PC | NVP loading: 0.891 mg cm^-2^  Rate: 2C/5C | 700/1000 | 95.58/95.31% | [52] |
| **DOL/DME/NaPF_6_/FEC** | **NVP loading: 2 mg cm^-2^**  **Rate: 2C/5C** | **2000/1100** | **93.7%/94.3%** | **This work** |

**Table S4** Electrochemical performance of full cells with various alloy anodes and SFC-Na

| Cathode | Anode | Cycle life | Rate | Capacity retention | Anode reaction mechanism | Refs. |
| --- | --- | --- | --- | --- | --- | --- |
| Na_x_Fe[Fe(CN)_6_] | Na-Au alloy | 300 cycles | 1C | 85.7% | Alloying | [53] |
| Na_4_Fe_3_(PO_4_)_2_ | Na-K/C alloy | 200 cycles | 1C | 94.6% | Alloying | [54] |
| Na_3_V_2_(PO_4_)_3_ | Na-Bi alloy | 1000 cycles | 5C | 90% | Alloying | [55] |
| Na_3_V_2_O_2_(PO_4_)_2_F | Na-In/C alloy | 800 cycles | 1C | 87.6% | Alloying | [56] |
| Na_3_V_2_(PO_4_)_3_ | Sb/GO | 50 | 1.5C | 81.3% | Alloying | [57] |
| Na_3_V_2_(PO_4_)_3_ | Sb@HCF | 1000 | 1C | 84.1 | Alloying | [58] |
| **Na_3_V_2_(PO_4_)_3_** | **SFC-Na** | **3000 cycles** | **2C** | **92.2%** | **Surface-induced “top” Na deposition** | **This work** |
|  |  | **1100 cycles** | **5C** | **94.3%** |  |  |
